# Modeling and Optimization of Extruded Corn Product Fortification

**DOI:** 10.3390/foods15020208

**Published:** 2026-01-07

**Authors:** Jelena Filipović, Ivica Djalovic, Milenko Košutić, Milica Nićetin, Biljana Lončar, Miloš Radosavljević, Vladimir Filipović

**Affiliations:** 1 Institute of Food Technology, University of Novi Sad, Bulevar Cara Lazara 1, 21000 Novi Sad, Serbia; jelena.filipovic@fins.uns.ac.rs (J.F.);; 2 Institute of Field and Vegetable Crops, National Institute of the Republic of Serbia, Maxim Gorki 30, 21000 Novi Sad, Serbia; 3 Faculty of Technology Novi Sad, University of Novi Sad, Bulevar Cara Lazara 1, 21000 Novi Sad, Serbiamilos1506@gmail.com (M.R.);

**Keywords:** extrusion technology, corn-based snacks, quinoa fortification, gluten-free extrudates, response surface methodology, screw speed, nutritional enhancement, physical and sensory properties, Z-score optimization, pseudocereals

## Abstract

The present study aimed to model and optimize the fortification of corn-based extruded flips with quinoa flour to improve their nutritional, functional, and sensory quality while maintaining desirable technological properties. Corn flour was partially replaced with quinoa flour at levels of 0, 10, 20, and 30%, and the mixtures were processed using a twin-screw extruder at three screw speeds (350, 500, and 650 rpm). The influence of formulation and mechanical energy input on product quality was evaluated through comprehensive characterization, including bulk density, expansion index, texture, color, chemical composition, mineral profile, amino acid and fatty acid composition, and descriptive sensory attributes. Response surface methodology (RSM) was applied to model the effects of quinoa addition and screw speed on 56 quality responses. The Z-score approach was employed to identify optimal processing conditions. The results showed that from a technological and nutritional perspective, formulations containing 20–30% quinoa processed at medium to high screw speeds (500–650 rpm) provided the most balanced products. Z-score optimization identified that the sample with 20% quinoa extruded at 650 rpm showed a balanced combination of enhanced nutritional characteristics and preserved physical and sensory quality.

## 1. Introduction

Extruded products are a popular group of ready-to-eat snacks due to their convenience, texture, and sensory appeal, but unlike classic cereal-based snacks, which are typically low in nutrient density and high in calories and fat, they can offer a more beneficial nutritional profile [1,2]. The extrusion process, which involves short-term heat treatment at high temperatures, destroys harmful microorganisms, antinutrients, and enzymes, producing extruded snacks with improved digestibility of proteins and starches, retained micronutrients, and an extended shelf life [3]. The use of pseudocereals such as buckwheat, amaranth, and quinoa further enriches extruded products with proteins, soluble dietary fibers, and essential amino acids that are limited in classic grain snacks [4,5]. These products can be formulated as gluten-free options, offering an eco-friendlier alternative, since pseudocereals require fewer resources for cultivation compared to conventional crops [6,7]. In this context, there is a significant opportunity to develop innovative, healthier, and nutritionally enhanced extruded snacks that combine convenience with superior dietary benefits [2,8].

Quinoa (*Chenopodium quinoa* Willd.) has gained global popularity due to its exceptional nutritional profile, genetic diversity, and adaptability to a wide range of growing conditions [5,9]. Native to the Andean region of South America, quinoa is a pseudocereal that offers a rich source of protein, essential amino acids, dietary fiber, vitamins, and minerals, high-quality fatty acids, antioxidants, and other important multiple bioactive compounds [4,10]. Quinoa flour, a gluten-free ingredient often used in breads, crackers, and cookies, combines high protein and bioactive compound content with an excellent amino acid profile, including high levels of lysine, glutamic, and aspartic acids, lower levels of proline and arginine than other grains, and good bioavailability [3,7].

Quinoa is proving to be a promising, high-quality crop in Serbia that can successfully adapt to local climatic conditions [11]. Research has demonstrated that different varieties, such as Puno and Titicaca, can be profitable with a careful cost–benefit analysis, while proper agrotechnical practices and adequate irrigation enable optimal growth and high yields [12,13]. The results have shown that quinoa in typical agroclimatic conditions of Serbia has a higher content of protein and most essential amino acids, especially lysine and methionine, compared to wheat. In addition, quinoa contains significant amounts of vitamins, including thiamine and vitamin C, and is naturally gluten-free, all of which are sought-after properties on the domestic and global markets [11,12].

Extrusion allows for the modification of the physical properties of snack products, including their density, texture, porosity, and shape. The expansion index, a key factor in snack quality, is primarily influenced by the composition and the moisture level of the formulation, as well as by operating conditions [1,7]. The expansion and texture of extruded foods are inversely related to the protein and fiber content. Incorporating quinoa into the formulation improves the nutritional profile, increasing the protein, fiber, and mineral content, but may result in a denser texture and reduced expansion of the finished product [3,8,14]. Quinoa starch has a smaller granule size, lower amylose, and higher amylopectin content, compared with many common cereals, which together influence its digestibility, textural properties, and expansion behavior during the extrusion process [10]. By controlling the extrusion parameters, it is possible to optimize the balance between physical characteristics and nutritional values of gluten-free extruded snacks with quinoa [3].

Despite an increasing number of studies on quinoa-based extruded products, there is a notable lack of studies focusing on the extrusion behavior and product quality of snacks formulated with quinoa cultivated in Serbia. Given that the chemical composition and functional properties of quinoa are strongly influenced by genotype and growing environment, it is essential to evaluate how locally produced Serbian quinoa performs during extrusion processing and how mechanical energy input affects the final product characteristics.

Therefore, the objective of this study was to optimize the production of quinoa-based extruded products using quinoa grown in Serbia by investigating the effect of different levels of mechanical energy on the physical, technological, and functional properties of the final extruded products.

## 2. Materials and Methods

### 2.1. Material

The raw materials used in this research were obtained from the following:

- Corn (*Zea mays*): cultivar NS 3022 (FAO 360), Institute of Field and Vegetable Crops, harvested in the year 2025, Novi Sad, Serbia.
- Quinoa (*Chenopodium quinoa* Willd.): cultivar KVL 52, grown by local producer in Bačka Palanka, Serbia, cadastral no. 37/5, Bačka Palanka 2, harvested in the year 2025.

Characterization of the used raw materials’ chemical and mineral composition, particle size, and color characteristics is presented in Table 1.

### 2.2. Sample Preparation

Quinoa was ground on a Roskamp Champion (Waterloo, IA, USA) device, with 3 pairs of rollers. Then, a mixture of corn flour with different quinoa flour substitutions of 0%, 10%, 20%, and 30% was prepared.

Corn flour and quinoa flour were weighed on a laboratory scale in the following ratios:

- A ratio of 10:0 = corn flour/quinoa flour;
- A ratio of 9:1 = corn flour/quinoa flour;
- A ratio of 8:2 = corn flour/quinoa flour;
- 7:3 = corn flour/quinoa flour.

After weighing corn and quinoa in certain ratios, the samples were homogenized in a Forberg model F-6-RVC twin-shaft paddle mixer (Forberg International AS, Oslo, Norway), with a volume of 6 L. The mixing time, or homogenization, was t = 90 s. The moisture content of each blend was adjusted to 17% by adding water during mixing. The moisture content of the blends was determined using a moisture analyzer.

To test the impact of mechanical and thermal energy on the physical, chemical, nutritional, and functional properties of extruded products fortified with quinoa, three different screw speeds were used: 350, 500, and 650 rpm. The experimental plan with varied levels of quinoa flour substitution and screw speed is presented in Table 2:

### 2.3. Extrusion Process

Samples were produced using a rotating twin-screw extruder Bühler BTSK 30/28D (Bühler Holding AG, (Uzwil, Switzerland)) with 7 sections, and a length/diameter ratio = 28:1. The screws are interlocked and rotate in the same direction. The extruder barrel length was 880 mm in total. The extruder had two quenching tools for heating/cooling the parts. The first tool had the quenching water temperature set at 60 °C for zone 1, and the second tool had the water temperature set at 140 °C for zones 6–7. A die was used that had a single 4 mm diameter opening with a conical inlet (total die opening was 12.56 mm^2^). A screw configuration specifically designed to produce directly expanded extruded products was used. The feed rate was 25 kg/h, and the screw speed was varied at three levels: 350, 500, and 650 rpm. In order to specifically determine the impact of screw speed change on the extrusion process and the resulting behavior of the materials, the values of the other process factors, the barrel temperatures, and the feeding rates remained constant in all the experiment designs.

**Figure 1:**
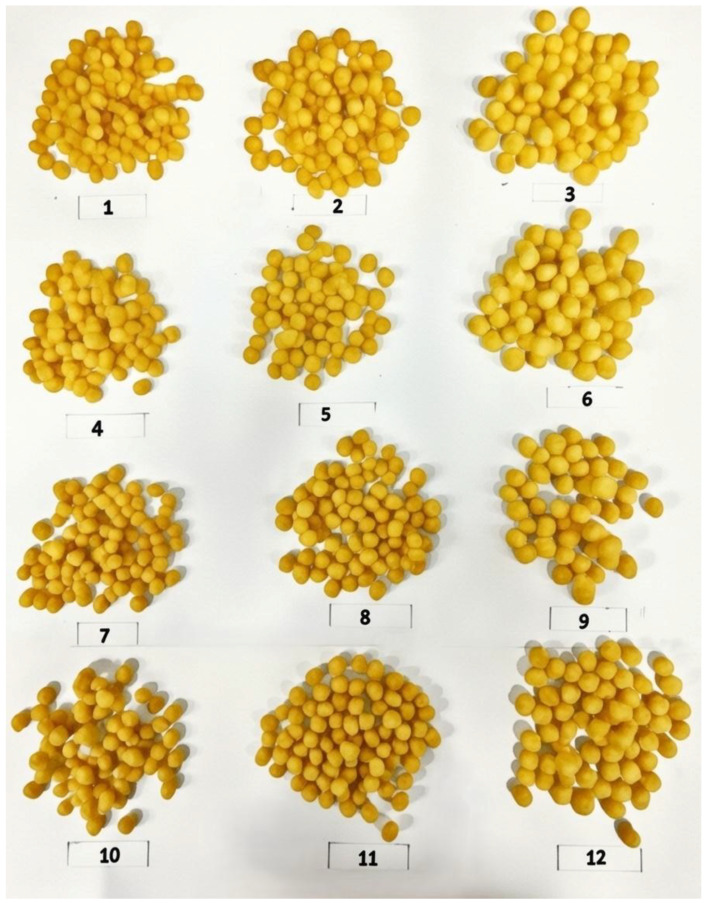
Visual appearance of extruded products produced with different quinoa substitution levels and screw speeds. Sample codes correspond to specific formulations and processing conditions described in Table 2.

A water pump was used to add water directly to the feed section of the extruder assembly, allowing changes in the material moisture content. Pressure and die temperature sensors were placed at the material outlet. Extrusion data are as follows: a die temperature of 150 °C; a die pressure of 0.58 bar; a motor load of 5.52 kW; and a specific mechanical energy of 156.70 Wh/kg were read directly from the extruder PLC screen. To achieve the final product length, the cutter at the exit of the extruder die was equipped with six knives, with a rotation speed set at 450 rpm. The extruded products were tested for their physical, technological, and functional characteristics 72 h after production. The extruded samples are shown in Figure 1.

### 2.4. Extruded Products Characterization

#### 2.4.1. Bulk Density

Bulk density (BD) of extruded products was calculated as the ratio of the extruded product’s mass and cylinder volume [15]. Six samples were used for each extruded product to calculate the mean.

Bulk density = weight of extruded product (kg)/Cylinder volume (m^3^)

#### 2.4.2. Expansion Index

Expansion index (EI) was calculated as the ratio of the extruded product and die diameters, according to Lu [16]. Six samples were used for each extruded product to calculate the mean.

### 2.5. Texture Properties

The textural properties of the extruder products were tested using a TA-XT2 Texture Analyzer (Stable Micro System, Godalming, UK) according to a modified method (dry cat food P35). The testing method involved placing 3 extruded products from each sample, oriented perpendicular to the probe, on a flat surface of the device, and applying compression using a cylindrical stainless steel probe with a diameter of 45 mm (P45) at a load cell of 50 kg and a breaking force of 100 g. The settings during the test were as follows: the speeds before the test, during the test, and after the test were set to 2.00, 1.00, and 10.00 mm s^−1^, respectively. The product hardness was defined as the force required to achieve the first product fracture, read as the maximum value from the obtained force–probe path curve (N). Measurements of the textural characteristics of the extruded products were performed in six replicates, 72 h after production, on each batch of flakes in 12 samples, at a temperature of 25 °C.

### 2.6. Color Instrumental Analysis

Color parameters of raw material and extruded product samples 72 h after production were determined in six replications, using a Chroma Meter (CR-400, Konica, Minolta, Tokyo, Japan) tristimulus colorimeter (contact surface diameter was 8 mm). Before measuring the samples, calibration was performed using a white color standard. The color parameter results were presented according to the CIElab color system, where the coordinates are defined as follows: L*—lightness (from 0 (black) to 100 (white)); a*—greenness/redness (from −a* (green) to +a* (red)); and b*—blue/yellowness (from −b* (blue) to +b* (yellow)) [17].

The color variation between the control sample and the quinoa-fortified samples (∆E) is given by Equation (1):

∆E = (∆L^2^ + ∆a^2^ + ∆b^2^)1/2 (1)

where ∆L is the difference in parameter L between flips with 0% quinoa and the extruded quinoa-fortified product, ∆a is the difference in parameter a between the control and the extruded quinoa-fortified product, and ∆b is the difference in parameter b between the control and the extruded quinoa-fortified product.

### 2.7. Proximate Analysis

An approximation of the chemical composition of raw material and extruded samples was conducted according to AOAC standard methods [18]: moisture content (method No. 934.01), protein content (method No. 950.36), starch content (method No. 996.11), total sugars content (method No. 2020.07), total carbohydrates content (method No. 986.25), lipid content (method No. 935.38), cellulose content (method No. 973.18), ash content (method No. 930.22), and total dietary fiber (method No. 985.29). Each measurement was performed in three replications.

### 2.8. Minerals Analysis

The mineral contents of zinc (Zn), copper (Cu), iron (Fe), potassium (K), magnesium (Mg), calcium (Ca), manganese (Mn), and sodium (Na) of raw material and extruded products were determined in accordance with the standard methods of AOAC [18]. Minerals were determined by atomic absorption spectrophotometry (method No. 984.27) on a Varian Spectra AA 10 (Varian Techtron Pty Ltd., Mulgrave, Melbourne, VIC, Australia). Each measurement was performed in three repetitions.

### 2.9. Amino Acids

The amino acid composition of whole cakes and their fractions was determined by ion-exchange chromatography using an automatic amino acid analyzer (Biochrom 30+, Biochrom, Cambridge, UK), following the method described by Rakita et al. [19]. Amino acids were separated on a strong cation-exchange column and derivatized with ninhydrin, with absorbance measured at 570 nm, except for proline, which was detected at 440 nm. Identification was carried out by comparing retention times with those of a standard amino acid mixture (Sigma-Aldrich, St. Louis, MI, USA). The results were expressed as g of amino acid per 100 g of sample.

### 2.10. Fatty Acid Analysis

Lipids were isolated from the samples by cold extraction, performed at ambient temperature without external heating, using a chloroform–methanol mixture (2:1, *v*/*v*) for 2.5 h. The obtained lipid extracts were subsequently derivatized to fatty acid methyl esters (FAMEs) and analyzed by gas chromatography using an Agilent 7890A system equipped with a flame ionization detector (Agilent Technologies, Santa Clara, CA, USA). Chromatographic separation was achieved on a fused-silica capillary column SP-2560 (100 m × 0.25 mm i.d., 0.20 μm film thickness; Supelco, Bellefonte, PA, USA) under the conditions reported [20,21]. Individual FAMEs were identified by comparison with an authenticated standard mixture (Supelco 37 Component FAME Mix, Sigma-Aldrich, St. Louis, MI, USA), and the results were expressed as g/100 g of fat.

### 2.11. Descriptive Sensory Analysis (Color, Shape, Hardness, Crispiness, Expansion Perceptions, and Taste)

Extruded products’ sensory profiling was performed by a panel of ten experienced food product evaluators employed on Faculty of Technology, Novi Sad, and the Institute of Food Technology, using the appropriate standard—ISO 6658:2017 [22].

Selection of initial sensory profiling descriptors was performed by the leading evaluator for the extruded product samples, using the descriptive sensory method. Subsequently, the descriptors were finely adjusted by the rest of the evaluators for a better definition of extruded products’ sensory profiles.

Descriptors for extruded products: color, shape, hardness, crispiness, expansion perception, and taste were included in the final extruded products’ descriptive sensory analysis form, as shown in Supplementary Materials, Table S1.

Each descriptor intensity was defined by a 9-point scale, where the lowest intensity was marked with 1, and the highest intensity was marked with 9 (ISO 4121:2003) [23], except for descriptors for color, hardness, and taste, where optimal values were set to 5, and absolute deviation from this value was used for further calculations.

Descriptive sensory analysis was performed 72 h after samples’ production, in the laboratory designated for sensory analysis in the Institute of Food Technology, Novi Sad, Serbia. The laboratory is designed following the ISO 8589:2007 [24] standard. Panelists were first required to read and fill out the informed consent form integral part of the extruded products’ descriptive sensory analysis form, as shown in Supplementary Materials, Table S2, which, by its content, follows the Declaration of Helsinki guidelines, and after their formal acknowledgment, the analysis began. Panelists were served with the extruded products’ samples for testing on white plastic plates, which were coded with random three-digit numbers obtained from the table of random numbers. The evaluators were provided with enough water to rinse their mouths between tasting each sample.

### 2.12. Methods of Statistical Analysis

#### 2.12.1. Response Surface Methodology

Response surface methodology (RSM) was selected to estimate the effects of quinoa quantity addition and extruder screw speed on extruded product quality parameter responses. The accepted experimental design was according to Box and Behnken’s full factorial design, already applied to similar new food product designs [25]. The independent variables were quinoa quantity addition (X_1_) of 0%, 10%, 20%, and 30%, and screw speed (X_2_) of 350 rpm, 500 rpm, and 650 rpm. The observed dependent variables were the extruded products’ quality parameter responses of physical and technological characteristics: BD, EI, Har, NF, and Crw (Y_1_–Y_5_, respectively); instrumental color characteristics: L*, a*, b*, and ΔE (Y_6_–Y_9_, respectively); chemical composition: moisture, proteins, starch, total sugars, total carbohydrates, lipids, cellulose, ash, and total dietary fiber (Y_10_–Y_18_, respectively); mineral matter composition: Zn, Cu, Fe, K, Mg, Ca, Mn, and Na (Y_19_–Y_26_, respectively); essential amino acids’ content: isoleucine, leucine, lysine, methionine + cystine, phenylalanine + tyrosine, threonine, tryptophan, valine, and total essential amino acids (Y_27_–Y_35_, respectively); non-essential amino acids’ content: alanine, arginine, aspartic acid, glutamic acid, glycine, histidine, proline, serine, and total non-essental amino acids (Y_36_–Y_44_, respectively); fatty acids content: palmitic, stearic, oleic, linoleic, linolenic and total fatty acids (Y_45_–Y_50_, respectively); and descriptive sensory analysis: color, shape, hardness, crispiness, expansion perception, and taste (Y_51_–Y_56_, respectively).

A model was fitted to the response surface generated by the experiment. The following second-order polynomial model was fitted to the data. Models of the following form were developed to relate fifty-six responses (Y_k_) to two process variables (X_i_):

(2) $$Y_{k}=\beta_{k0}+\sum_{i=1}^{2}\beta_{ki}\cdot X_{i}+\sum_{i=1}^{2}\beta_{kii}\cdot {X_{i}}^{2}+\beta_{k12}\cdot X_{1}\cdot X_{2}\;k=1–56$$

where β_k0_, β_ki_, β_kii_, and β_k12_ are constant regression coefficients; Y are extruded products’ quality parameter responses; X_1_ = coded quinoa addition level, and X_2_ = coded screw speed.

#### 2.12.2. Z-Score Analysis

Z-score analysis converts extruded products’ quality responses, via min–max normalization, into a dimensionless scale ranging from 0 to 1, allowing direct comparison of different responses and further statistical evaluation. The highest total Z-score, derived from the defined mathematical combination of all segment Z-scores, denotes the optimal extruded products’ overall quality profile [26].

Segment Z-scores are calculated as follows:

Extruded products’ physical and technological characteristics:

(3) $$S_{1i}=\frac{\sum_{k=1}^{2}\left(1-\frac{x_{ki}-x_{kmin}}{x_{kmax}-x_{kmin}}\right)+\sum_{j=1}^{3}\left(\frac{x_{ji}-x_{jmin}}{x_{jmax}-x_{jmin}}\right)}{5}$$

where *x_k_* are BD and Crw, and *x_j_* are EI, Har, and NF.

Extruded products’ instrumental color characteristics:

(4) $$S_{2i}=\frac{\sum_{l=1}^{3}\left(\frac{x_{li}-x_{lmin}}{x_{lmax}-x_{lmin}}\right)+\left(1-\frac{x_{mi}-x_{mmin}}{x_{mmax}-x_{mmin}}\right)}{4}$$

where *x_l_* are L*, a*, and b*, and *x_m_* is Δ E.

Extruded products’ chemical composition:

(5) $$S_{3i}=\frac{\sum_{n=1}^{2}\left(1-\frac{x_{ni}-x_{nmin}}{x_{nmax}-x_{nmin}}\right)+\sum_{o=1}^{7}\left(\frac{x_{oi}-x_{omin}}{x_{omax}-x_{omin}}\right)}{9}$$

where *x_n_* are moisture and lipids, and *x_o_* are proteins, starch, total sugars, total carbohydrates, cellulose, ash, and total dietary fiber.

Extruded products’ mineral matter composition:

(6) $$S_{4i}=\frac{\sum_{p=1}^{7}\left(\frac{x_{pi}-x_{pmin}}{x_{pmax}-x_{pmin}}\right)+\left(1-\frac{x_{ri}-x_{rmin}}{x_{rmax}-x_{rmin}}\right)}{8}$$

where *x_p_* are Zn, Cu, Fe, K, Mg, Ca, and Mn, and *x_r_* is Na.

Extruded products’ essential amino acids’ content:

(7) $$S_{5i}=\frac{\sum_{s=1}^{9}\left(\frac{x_{si}-x_{smin}}{x_{smax}-x_{smin}}\right)}{9}$$

where *x_s_* are isoleucine, leucine, lysine, methionine + cystine, phenylalanine + tyrosine, threonine, tryptophan, valine, and total essential amino acids.

Extruded products’ non-essential amino acids’ content:

(8) $$S_{6i}=\frac{\sum_{t=1}^{9}\left(\frac{x_{ti}-x_{tmin}}{x_{tmax}-x_{tmin}}\right)}{9}$$

where *x_t_* are alanine, arginine, aspartic acid, glutamic acid, glycine, histidine, proline, serine, and total non-essential amino acids.

Extruded products’ fatty acid content:

(9) $$S_{7i}=\frac{\sum_{u=1}^{3}\left(1-\frac{x_{ui}-x_{umin}}{x_{umax}-x_{umin}}\right)+\sum_{f=1}^{3}\left(\frac{x_{vi}-x_{vmin}}{x_{vmax}-x_{vmin}}\right)}{6}$$

where *x_u_* are palmitic, stearic, and total fatty acids, and *x_v_* are oleic, linoleic, and linolenic fatty acids.

Extruded products’ descriptive sensory analysis:

(10) $$S_{8i}=\frac{\sum_{w=1}^{3}\left(1-\frac{x_{wi}-x_{wmin}}{x_{wmax}-x_{wmin}}\right)+\sum_{x=1}^{3}\left(\frac{x_{xi}-x_{xmin}}{x_{xmax}-x_{xmin}}\right)}{6}$$

where *x_w_* are the absolute deviation of color, hardness, and taste values from optimal ones (value of 5), and *x_x_* are shape, crispiness, and expansion perception.

Total quality of extruded products’ Z-score:

(11) $$S_{i}=\left(0.2\cdot {S_{1}}_{i}+0.05\cdot {S_{2}}_{i}+0.15\cdot {S_{3}}_{i}+0.10\cdot {S_{4}}_{i}+0.15\cdot {S_{5}}_{i}+0.05\cdot {S_{6}}_{i}+0.15\cdot {S_{7}}_{i}+0.15\cdot {S_{8}}_{i}\right)$$

where extruded products’ nutritive (S_3i_ to S_7i_) and technological (S_1i_, S_2i_, and S_8i_) quality characteristics Z-score values contributed with 60% and 40% of significance to the total Z-score, respectively.

(12) $$\max\;[S_{i}]\to optimum$$

Analysis of variance (ANOVA) and RSM were performed using StatSoft Statistica, for Windows, ver. 12 program. The model was obtained for each dependent variable (or response), where factors were rejected when their significance level was less than 95%. Z-score values were calculated using Microsoft Excel ver. 2016 (Microsoft Corporation, Redmond, WA, USA).

## 3. Results and Discussion

The quinoa used in this research is locally grown in Serbia and the results of the analysis showed a high-quality compositional profile, usually associated with quinoa—elevated protein (17.14%), lipids (4.10%), and dietary fiber contents (7.30 g/100 g)—and a mineral-dense composition, particularly with high K and Mg content and significant Fe, Zn and Mn content, relative to corn. Obtained chemical and mineral matter content values fall within reported global ranges for quinoa and are comparable to nutrient-dense cultivars reported in the literature [8,27], confirming that Serbian-grown quinoa is a suitable raw material for nutritional fortification of extruded products.

The physical and technological characterization of extruded products provides valuable insight into how formulation and processing conditions affect product structure, texture, and overall quality. The selected parameters—bulk density (BD), expansion index (EI), hardness (Har), number of fractures (NF), and crispiness work (Crw)—are particularly sensitive to both mechanical energy input and raw material composition, serving as reliable indicators of extrusion performance [28]. BD reflects the compactness of the extrudate and is influenced by melt viscosity, air incorporation, and bubble stability during extrusion. EI is directly related to gas expansion and matrix elasticity, which are affected by starch gelatinization, protein network formation, and moisture content. Har, NF, and Crw collectively describe textural properties: higher mechanical energy and shear can thin cell walls, increase porosity, and promote crispiness, whereas protein–lipid interactions and fiber content can reinforce the matrix, increasing hardness and fracture resistance. By analyzing these parameters together, one can infer how extrusion variables—such as screw speed and pseudocereal fortification—interact with the raw material composition to shape the physical structure and textural properties of the final product.

The results presented in Table 3 demonstrated that both varied parameters: quinoa quantity addition and extruder screw speed statistically significantly affected all extruded products’ physical and textural characteristics. Increasing screw speed from 350 to 650 rpm statistically significantly reduced BD and increased EI values at all quinoa addition levels, with a BD reduction of a maximal of 70.39% and an EI increase of 46.40%. These results are in correlation with fundamental extrusion principles, where higher mechanical input intensifies starch gelatinization and greater melt temperature, facilitating vapor flashing and bubble growth at the die [29], while quinoa incorporation into the corn matrix intensified these effects, particularly at higher screw speeds. Extruded products’ instrumental color characteristics, depending on different quinoa quantity additions and extruder screw speeds are given in Table 4. Quinoa’s chemical profile, rich in proteins and unsaturated lipids (Table 5), probably influenced melt rheology, favorizing cell expansion under sufficient energy input. Similar mechanisms of expansion due to pseudocereal incorporation have been reported [30].

Textural parameters (Har, NF, and Crw) showed similar trends, as in the case of BD and EI. Har decreased statistically significantly with increasing quinoa quantity addition and screw speed, lowering this response for 66.72% at maximal levels of varied parameters. Higher extruder screw speeds probably produced an extrudate structure characterized by thinner cell walls and larger pores, resulting in reduced fracture force and improved crispiness. This proposition is supported by the observed values of statistically significantly increased NF and Crw, indicating the development of a friable, aerated matrix, which is characteristic of highly expanded snack products [31].

The decrease in Crw values, with the increased quinoa quantity addition, may originate from the disruption of the continuous starch matrix by quinoa-rich proteins and lipids, producing a more fragile microstructure [32].

The interaction between two varied parameters—quinoa fortification and mechanical energy input—appears to be synergistic, since the most desirable physical attributes of the extruded products were consistently observed at high levels of these two varied parameters.

One of the key factors influencing customer favorability of different food products is its color, since it directly affects the consumer perception and acceptability [33]; hence, it is an inevitable parameter to be investigated in new product type development.

In Table 4, the results of the extruded products’ color characteristics are presented. All tested quinoa-fortified extruded products’ color responses were affected by both varied parameters, where quantity addition showed a statistically significant effect, at the level of significance of *p* < 0.05. Increasing quinoa fortification level led to a progressive reduction in L* values (in the maximal extent of ~3% value decrease). Inherent darker natural pigment profile, together with higher protein and lipid content of quinoa (Table 5), can contribute to more significant Maillard and browning reactions during extrusion [34].

Higher quinoa addition levels led to the shift of a* values toward a negative scale, indicating an increase in green tonalities of the extruded products, possibly attributed to the phenolic compounds content and the inherent coloration of quinoa flour [35].

The reduction in b* values with the increasing quinoa fortification levels (for 17.89% in the maximal extent) reflects a reduction in the characteristic yellow hue of the corn-based extrudate products, probably due to the diminishing the carotenoids content and altered starch–protein interactions affecting pigment stability under thermal and mechanical stress [36,37].

Second, the varied parameters and screw speeds showed a more nuanced effect on all extruded products’ color characteristics. Higher screw speeds increased L* and b* values and decreased a* values, within each quinoa fortification level, indicating that greater shear and energy input and more complete starch gelatinization favorized bubble formation and structural expansion, producing lighter matrices, with more of a yellow tone that reflects more light [38].

Extruded products’ ΔE values statistically significantly increased with a quinoa-level addition to values exceeding 7.0 at 30% of quinoa addition. These ΔE values surpass the commonly cited threshold for perceptible color differences in extruded snacks (ΔE > 3), highlighting that quinoa incorporation has a visually apparent impact on final product appearance [33].

Extrusion process parameters can significantly affect the final product’s nutritional content and other quality attributes [39]. Maintaining and enriching the nutritional quality of foods during extrusion processing are the main research concerns [40].

In Table 5, the results of the extruded products’ chemical composition are shown, from which the moisture content decreased together with increasing screw speeds and quinoa addition levels (the highest moisture content decrease was 0.61 percentile point—sample 12 in comparison to the control sample (1)). These results can be justified by intensified mechanical energy input and enhanced starch gelatinization, which leads to more efficient water evaporation during extrusion. This moisture range remained typical for expanded snack products, indicating that quinoa addition did not compromise extruded product stability [41].

Extruded products’ protein content statistically significantly increased with the increasing level of quinoa fortification, enchanting final product protein content for 2.35% in comparison to the control sample, reaching a level of nearly 10% (at 30% quinoa fortification). Quinoa’s high protein content and its inherent structural resilience during extrusion provided a positive effect on final product protein content [42].

Screw speed, as a varied parameter, did not statistically significantly lower extruded products’ protein levels within each quinoa addition level group, suggesting that the used extrusion process parameters did not induce significant protein denaturation or degradation.

In contrast, extruded products’ starch content consistently decreased with both varied parameters, where the effect of screw speed was statistically significant. Quinoa, as a raw material, contains less starch than corn, while the reduction at elevated screw speeds can be attributed to increased starch breakdown and dextrinization caused by higher shear and energy input. The lowest extruded products’ starch content values were observed at 650 rpm for all quinoa fortification levels, which aligns with the expected increase in mechanical degradation of starch granules. These results are consistent with literature data, where it is stated that extrusion induces starch molecular fragmentation and reduces ordered crystalline structures [43].

Extruded products’ total sugar content showed a slight reduction with increasing screw speed, likely due to their participation in Maillard reactions and consequent sugar degradation during high-energy processing [44]. Quinoa addition did not statistically significantly lower extruded products’ sugar content, due to slightly lower initial content than corn flour (Table 5).

Total carbohydrate content values for extruded products statistically significantly decreased with increasing quinoa levels, since quinoa, as a raw material, contributes more proteins, fats, and fiber content relative to starch-rich corn grits. As screw speed increased, carbohydrate values within each group changed but were not statistically significant, remaining relatively stable, since the screw speed affects starch transformation [43], but not the proportion of carbohydrates inherently present in the products’ formulation. The highest decline in total carbohydrate content values was observed in sample 12 (with 30% of quinoa addition, at the 650 rpm of the screw speed), and it was for 3.17% in comparison to the control sample (1).

A consistent trend of statistically significant increase for lipids, cellulose, ash, and total dietary fiber content of extruded products with every quinoa fortification increment was noted. These results originate from quinoa’s richer nutritive profile, presented in Table 5, in comparison to the corn grits. Even at the 10% substitution level, this enrichment was evident. The other investigated parameter of screw speed not only statistically significantly affects lipids, cellulose, ash, and total dietary fiber content, suggesting that the increasing mechanical energy input did not degrade these compounds, but it may have also slightly concentrated them due to lower moisture retention.

The maximal increase in lipids, cellulose, ash, and total dietary fiber content was obtained at the highest levels of quinoa addition and screw speed of 500 rpm and 350 rpm only in the case of total dietary fiber content responses, and was as follows: 2.04 times, 19.80%, 2.50 times, and 11.40%, respectively.

These enhancements are nutritionally favorable, as mineral- and fiber-rich extruded snacks can contribute to improved digestive function and glycemic response. Screw speed had a mild decreasing effect on fiber content within each quinoa fortification level, which may reflect partial mechanical breakdown of insoluble fiber components at higher shear levels.

In Table 6, the results of the extruded products’ mineral matter content responses are presented. The presented results indicated that the mineral matter composition of the extruded products was strongly influenced by both quinoa fortification level and screw speed, reflecting quinoa’s inherently high micronutrient density (Table 6) and the known effects of thermomechanical processing on mineral retention and distribution [45]. Increasing the substitution level of corn grits with quinoa statistically significantly increased the extruded products’ concentrations of Zn, Cu, Fe, K, Mg, Ca, and Mn, consistent with quinoa’s superior mineral profile relative to corn and conventional cereals, as shown in Table 6 [46].

On the other hand, Na content decreased statistically significantly with increasing quinoa addition, reflecting the lower native Na level in quinoa compared with corn (Table 6).

The maximal increase in all mineral matter responses and decrease in case of Na was obtained at the highest levels of quinoa addition, while all screw speed levels exerted maximal results, depending of specific mineral (at the level of 350 rpm: Zn, Mg, Mn and Na; at the level of 500 rpm: K; and at the level of 650 rpm: Cu, Fe and Ca). The maximal obtained increase in Zn, Cu, Fe, K, Mg, Ca, and Mn was 2.46 times, 42.4%, 3.00 times, 2.48%, 2.56 times, 2.07 times, and 4.04 times, respectively, and the maximal obtained decrease for Na was 49.82%.

The effect of screw speed on mineral matter content was less pronounced than quinoa addition, but still increasing screw speed statistically significantly increased Zn, Fe, K, and Mg concentrations, and not statistically significant in cases of other mineral matter responses, likely due to greater moisture loss and a corresponding concentration effect, as previously proposed [45].

Non-essential amino acids are significant contributors to the nutritional and functional quality of extruded products. Their distribution reflects both raw material protein composition and extrusion-induced transformations that affect protein unfolding, Maillard reactivity, and overall product functionality [47,48]. Quinoa exhibits a well-distributed non-essential amino acid (NEAA) profile, characterized by relatively high contents of glutamic acid, aspartic acid, and alanine. In quinoa proteins, non-essential amino acids account for approximately 55–60% of total amino acids, resulting in a non-essential to essential amino acid ratio close to 1.2–1.4, which is comparable to or higher than that of common cereal proteins. This compositional feature provides an opportunity to enhance the non-essential amino acid content of traditional corn-based snacks [49].

Table 7 presents the extruded products’ non-essential amino acids’ content depending on two varied parameters. Extrusion at increasing screw speeds increased, in some cases, statistically significantly, the measured levels of all non-essential amino acids, at all quinoa addition levels. This outcome aligns with previously discussed mechanisms in the case of essential amino acids, where it was proposed that increased shear and mechanical energy input enhanced protein unfolding and improved amino acid extractability [36].

The increasing quinoa fortification levels produced a more complex effect, since quinoa protein profile significantly differs from the corn protein profile. Quinoa contains lower levels of proline and glutamic acid due to its albumin–globulin-rich profile, which contributes higher levels of arginine, histidine, and glycine [49]. This can explain the observed statistically significant increases in arginine, glycine, and histidine with increasing quinoa fortification levels, consistent with the characteristic quinoa amino acid profile. Conversely, alanine, glutamic acid, and proline content decreased statistically significantly with increasing quinoa fortification levels, reflecting the dilution of corn-based prolamins, which are naturally enriched in proline [48].

Total non-essential amino acids’ content showed statistically insignificant changes, with both increasing screw speeds and quinoa fortification levels. The contents of alanine, glutamic acid, proline, and total non-essential amino acids decreased 2.40%, 4.44%, 11.20%, and 2.17%, respectively, when the highest levels of screw speed and quinoa fortification were applied (sample 12) in comparison to the control sample (1). The contents of arginine, aspartic acid, glycine, histidine, and serine increased 26.99%, 8.50%, 12.05%, 10.98%, and 2.45%, respectively, when the same samples were compared as previously. At higher screw speeds, increased mechanical energy input and residence-time-dependent thermal stress can lead to protein denaturation and fragmentation, facilitating the release of free or low-molecular-weight amino acids such as glutamic acid and proline. Conversely, essential amino acids are more susceptible to degradation or participation in Maillard-type reactions under these conditions, resulting in an apparent increase in the relative contribution of non-essential amino acids to the total amino acids.

Although the absolute changes were less significant in comparison to essential amino acids, qualitative shifts regarding increases in arginine, histidine, and glycine content highlighted quinoa’s contribution to the extruded product’s functional and nutritional improvement.

Fatty acid composition is an important parameter of both the extruded products’ technological and nutritional quality, since it influences oxidative stability, shelf life, and health-related issues. Quinoa is known for its favorable lipid profile, compared with traditional cereals, making it a suitable raw material for enriching the nutritional quality of expanded products. Extrusion process parameters can affect the lipid availability and fatty acids’ distribution, highlighting the need to investigate the effect of process parameters on quinoa enrichment to shape the final fatty acid profile [48,49].

In Table 8, the results of extruded products’ fatty acid content are presented, from which the addition of quinoa increased the content of both saturated and polyunsaturated fatty acids. These trends are in correlation with the inherent quinoa lipid profile, containing higher levels of palmitic, staeric and especially linolenic acid, compared with conventional cereal raw materials [49].

Palmitic and stearic acid contents statistically significantly increased with the increasing quinoa fortification levels, regardless of the applied screw speeds. Furthermore, increasing screw speed increased these concentrations, although not statistically significant, probably due to enhanced lipid release caused by higher mechanical shear and cellular rupture during extrusion, a phenomenon commonly reported in the extrusion of oil-containing grains [48].

In the case of the oleic and linoleic acid content, a statistically significant decrease was observed with the increase in quinoa fortification levels, while the increase in screw speeds led to a statistically insignificant content increase. Their findings are in accordance with previously reported quinoa’s lower oleic and linoleic acid content, compared with corn [49].

The content of extruded products’ linolenic acid increased statistically significantly with increasing quinoa fortification levels, consistent with quinoa’s notably higher linolenic acid concentration [50]. The application of higher screw speed furthermore statistically significantly increased the extruded products’ linolenic acid content. This increase is nutritionally very significant, since most cereal-based snacks are poor sources of ω-3 fatty acids.

Total fatty acid content of extruded products statistically significantly increased with the increase in both varied parameters. The preservation of polyunsaturated fatty acids, at the screw speed of 650 rpm, indicated that the processing parameters did not exceed oxidative destruction limits, retaining fatty acid integrity in short-time high-temperature processes [48].

By the application of maximal values of varied parameters, the contents of palmitic, stearic, linolenic, and total fatty acids increased 17.62%, 29.17%, 2.67 times, and 7.68%, respectively, while contents of oleic and linoleic fatty acids decreased 3.70% and 5.96%, respectively, in comparison with the control sample (1).

The descriptive sensory analysis of new food products can help to identify the main sensory attributes that can be further addressed in the process of new product creation, by investigating the limits of possible incorporation of innovative raw materials, in the effort of food fortification [51].

**Figure 2:**
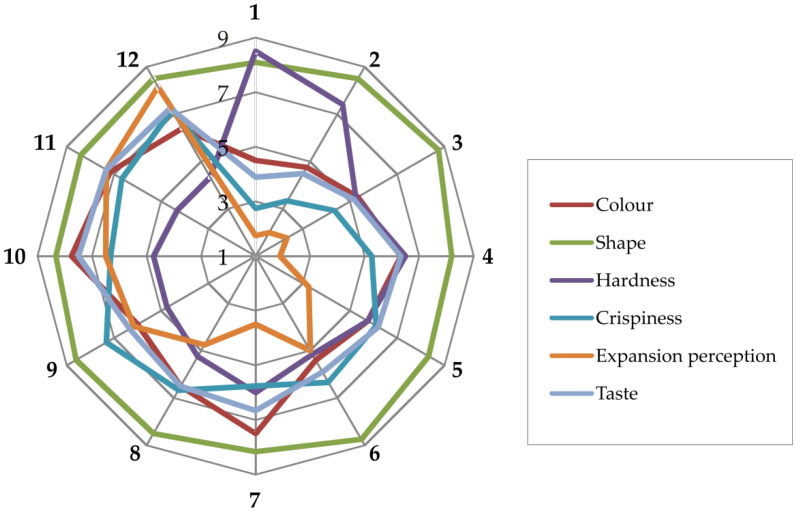
The sensory profile of samples 1–12 based on six attributes: color, shape, hardness, crispiness, expansion perception, and taste. Sensory evaluation was performed using a 9-point hedonic scale, and data are expressed as mean values.

The descriptive sensory analysis of extruded products, presented in Table S3 and Figure 2, revealed statistically significant effects of quinoa inclusion and screw speed on all descriptive sensory attributes of the extruded products.

Extruded products’ color intensity increased statistically significantly with increasing quinoa fortification levels, corresponding to quinoa’s intrinsic darker pigments and its greater protein content that increases Maillard browning during extrusion [34]. These subjective observations of the trained panelists are in accordance with instrumental color characteristics, presented in Table S3.

Sensory perception of obtained extruded products’ shape uniformity exhibited minimal, statistically insignificant variation across all varied parameters, indicating that quinoa addition and screw speed did not compromise structural formation during the expansion process.

Sensory perception of extruded products’ hardness decreased statistically significantly with both increasing quinoa fortification level and with increasing screw speeds, indicating a shift in perception from soft to hard. The closest mark to optimal (5.16) was assigned to sample 6. Sensory perception of crispiness statistically significantly increased (from tough to crispy perception) with increasing levels of both varied parameters. These effects reflect greater starch gelatinization, disruption, and air-cell formation under enhanced mechanical shear and heat transfer [52].

Extruded products’ expansion perception statistically significantly shifted from compact to porous marks with both quinoa fortification level increase and the screw speed increase, corresponding to the reports that the addition of pseudocereal proteins at moderate levels can positively influence expansion stability, when combined with sufficient shear [34].

The results obtained from subjective sensory perception of extruded products’ textural characteristics are in correlation with the instrumentally obtained textural results, as presented in Table S3, highlighting that the effect of a quinoa addition on products’ texture is significant enough to be subjectively perceived, not only instrumentally.

The relatively low sensory scores for crispiness and expansion perception in some extruded products can be attributed to both raw material composition and processing conditions. The addition of quinoa increases protein and fiber content, which can limit starch gelatiniation and reduce melt elasticity, leading to denser extrudates with smaller, less uniform air cells. Lower screw speeds and insufficient mechanical energy exacerbate this effect, resulting in reduced expansion and less pronounced crispiness. Conversely, higher screw speeds enhance shear and heat input, partially compensating for the structural constraints imposed by quinoa, which explains the gradual improvement in crispiness and expansion perception with increasing screw speed and quinoa levels up to a certain threshold.

Extruded products’ taste marks statistically significantly increased only with a quinoa fortification level increase, while screw speed increase exerted a statistically significant shift in taste, but only in samples without quinoa addition. The increase in taste marks above the optimal one (mark 5) indicated a taste shift towards a more bitter taste, indicating that the level of 30% quinoa fortification exerted significant taste modulation.

The RSM was used for developing mathematical models of extruded products’ quality parameters in dependence on quinoa level fortification and screw speed, as shown in Table 1. The RSM demonstrated excellent predictive performance for nearly all of the fifty-six evaluated extruded products’ quality parameters, as confirmed by the range of determination coefficients with high values: R^2^ = 0.90–0.99.

ANOVA revealed that the share of quinoa linear terms and, to a lesser extent, screw speed linear terms were the statistically significant contributors to variability across all quality response groups, while quadratic effects were generally weaker and/or statistically insignificant, Table S4. The share of quinoa linear terms, only in cases of number of fracurability, Δ E, total fatty acids, shape, and hardness, of fifty-six cases in total were not statistically significant in model formation, while the share of quinoa quadratic terms statistically significantly contributed on model formation in cases of crispier work, proteins, lipids, total non-essential amino acids and crispiness, indicating the profound effect of quinoa fortification on every quality parameter tested.

The trends of the effects of both varied parameters on developed mathematical models can be visually noticed in Figures S1–S8, where the graphic presentations of modelled dependence of all tested quality parameters from share of quinoa and screw speed are shown.

Screw speed linear term statistically significantly influenced formation of almost all physical and technological characteristics, except for number of factorability: on L*, moisture, proteins, starch and total sugar of chemical composition; on all of the essential and non-essential amino acids composition responses; and on all fatty acids composition responses except for linoleic fatty acid and on color, shape, crispiness and expansion perception, of the descriptive sensory analysis responses. Screw speed quadratic term was statistically significant only in cases of proteins, total sugars, phenylalanine + tyrosine, total essential amino acids, alanine, total non-essential amino acids, and palmitic fatty acid content.

The cross product of share of quinoa x screw speed statistically significantly contributed to the formation of the following quality responses models: bulk density, hardness, moisture, starch, lysine, valine, arginine, proline, color, and shape, hence indicating that the simultaneous influence of varied parameters was limited.

Residual variance was not statistically significant in any case of fifty-six modelled responses, indicating good fit quality of all developed mathematical models with the experimental data.

In Table S5, the regression coefficients of fifty-six second-order polynomial models of physical and technological, instrumental color characteristics, chemical, mineral matter, essential amino acids, non-essential amino acids, and fatty acids content, and descriptive sensory responses of the extruded products are shown. The statistical significance of individual coefficients is marked. These coefficients (β_k0_–β_k12_) can be used for completing quadratic equations that describe mathematical models of extruded products’ quality parameters. Based on complete equations and inputted desired values of quinoa-level fortification and screw speed (of the experimentally determined range), values of all fifty-six quality responses can be calculated, allowing for the prediction and precise adjustment of the final extruded product quality.

**Figure 3:**
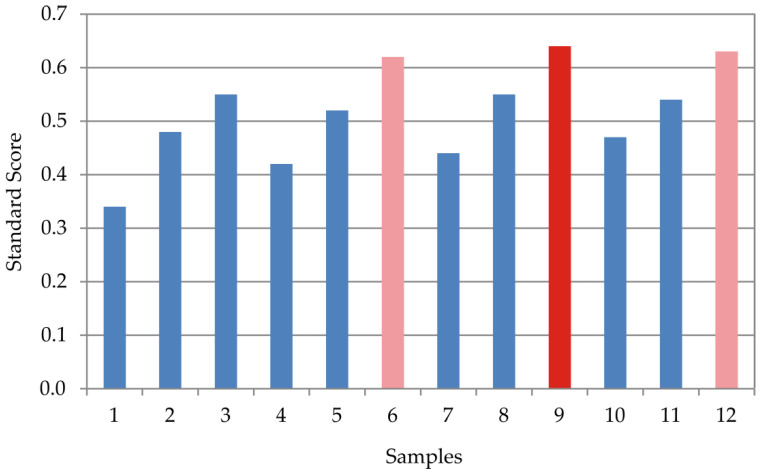
Evaluation of the samples 1–12 using Z-score methodology.

Z-score analysis was applied in an effort to investigate the extruded products’ segment quality characteristics (S1 to S8) and to define the optimal combination of applied parameters to obtain maximal overall quality characteristics (Total S), as shown in Table S6 and Figure 3.

The highest level of quinoa fortification caused the highest segment scores for physical and technological characteristics (S1), mineral matter composition (S4), and essential and non-essential amino acids composition (S5 and S6), while it had adversative effect on instrumental color characteristics (S2), fatty acid composition (S7), and descriptive sensory analysis (S8).

Total Z-Score values (Total S) were set to mathematically combine all segment quality characteristics in the following manner: nutritive significance and contribution to the Total S values with 60%; technological significance and contribution to the Total S values with 40%. Hence, the individual segment quality characteristics contribution was as follows: S1—20%, S2—5%, S3—15%, S4—10%, S5—15%, S6—5%, S7—15%, and S8 15%.

The maximal obtained Total S value of 0.64 was marked in extruded product sample 9 (20% of quinoa fortification level and screw speed of 650 rpm), as the extruded product sample with the maximal overall quality, combining the highest increase in the nutritive enrichment with the lowest technological quality decline.

## 4. Practical Implications

From an industrial perspective, the application of quinoa-fortified corn-based extrudates at the identified formulation range (up to 20% of quinoa addition and higher screw speeds) appears technically feasible within conventional single- or twin-screw extrusion systems commonly used in snack production. The observed improvements in expansion, texture, and nutritional profile were achieved without excessive deterioration of technological or sensory quality, indicating that quinoa can be incorporated at moderate levels without fundamentally altering process stability. The screw speed of 650 rpm, associated with favorable expansion and texture, is within the operational range of industrial extruders, suggesting that similar product characteristics could be reproduced at a larger scale, provided that specific mechanical energy input and moisture control are appropriately managed.

There are several limitations that should be considered when extrapolating laboratory-scale results to industrial production. Higher screw speeds and increased mechanical energy input may result in increased energy consumption and equipment wear, which could affect production costs and long-term operational efficiency. Also, quinoa flour has different flowability, particle size distribution, and lipid content compared with corn grits, which may influence feeding behavior, die pressure, and cleaning requirements during continuous operation. Variability in quinoa cultivar, growing conditions, and post-harvest processing could also introduce compositional variability, requiring raw material standardization to ensure consistent product quality.

The results of this study provide a scientifically supported basis for further pilot-scale trials, where process optimization, cost–benefit analysis, and shelf-life evaluation should be conducted to validate the commercial viability of quinoa-fortified extruded snacks.

## 5. Conclusions

The results of this study demonstrate that quinoa can be successfully incorporated into extruded flips at relatively high substitution levels (up to 30%) without compromising technological or sensory quality, provided that appropriate extrusion conditions are applied. High screw speeds ensured sufficient shear forces and melt homogeneity, enabling the production of nutritionally enriched snacks aligned with current clean-label and plant-based food trends. Nutritionally, the addition of quinoa significantly improved the protein, lipid, dietary fiber, and mineral content of extruded flips. Products containing 30% quinoa showed the highest nutrient density, while moderate screw speeds (350–500 rpm) favored the preservation of structural carbohydrates and dietary fiber. Quinoa enrichment also positively influenced protein quality by enhancing both essential and non-essential amino acid profiles. These findings confirm quinoa’s strong potential to improve amino acid balance in cereal-based extruded snacks. The incorporation of quinoa favorably modified the fatty acid composition of the extrudates, increasing α-linolenic acid content while maintaining desirable MUFA and PUFA profiles. Sensory evaluation indicated that quinoa enrichment, particularly at the 20% level, enhanced key sensory attributes, while higher screw speeds improved crispiness and expansion without negatively affecting flavor. Response surface methodology confirmed that quinoa level was the dominant factor influencing nutritional and quality attributes, whereas screw speed mainly contributed to physical structure and sensory texture. Z-score analysis suggested that the formulation containing 20% quinoa processed at 650 rpm represents a promising option, as it demonstrated balanced nutritional, technological, and sensory characteristics within the experimental range studied. These findings provide a strong scientific basis for the development of quinoa-enriched extruded snacks using locally adaptable quinoa varieties and optimized extrusion conditions.

## Figures and Tables

**Table 1:** Raw materials characterization.

	Corn Flour	Quinoa Flour
Chemical composition		
Moisture (%)	13.70 ± 0.14	10.61 ± 0.13
Protein (% d.m)	7.68 ± 0.09	17.14 ± 0.12
Starch (% d.m)	86.25 ± 0.96	72.26 ± 0.89
Total sugars (% d.m)	1.83 ± 0.01	1.71 ± 0.02
Total carbohydrates (% d.m)	90.93 ± 0.92	76.97 ± 0.90
Lipids (% d.m)	1.13 ± 0.03	4.10 ± 0.13
Cellulose (% d.m)	2.23 ± 0.03	2.62 ± 0.02
Ash (% d.m)	0.28 ± 0.01	1.60 ± 0.08
Total dietary fiber (g/100 g)	5.82 ± 0.06	7.30 ± 0.11
Mineral matter composition		
Zn (mg/kg)	4.50 ± 0.06	10.5 ± 0.81
Cu (mg/kg)	0.46 ± 0.02	4.01 ± 0.39
Fe (mg/kg)	6.95 ± 0.06	32.06 ± 0.98
K (mg/kg)	1107.04 ± 17.06	5468.46 ± 39.84
Mg (mg/kg)	235.52 ± 1.84	1092.71 ± 10.09
Ca (mg/kg)	26.42 ± 0.24	105.63 ± 1.97
Mn (mg/kg)	1.72 ± 0.02	7.37 ± 0.91
Na (mg/kg)	227.76 ± 1.97	71.11 ± 1.08
Particle size		
>350 µm	83.80 ± 1.81	88.19 ± 2.01
250–350 µm	9.02 ± 0.57	6.34 ± 0.43
150–250 µm	3.94 ± 0.20	3.44 ± 0.17
<150 µm	3.10 ± 0.21	2.38 ± 0.18
Color characteristics		
L*	80.93 ± 2.99	73.74 ± 0.71
a*	4.24 ± 0.39	1.84 ± 0.21
b*	32.37 ± 2.84	14.09 ± 2.01

**Table 2:** Experimental designs to produce flips products with different levels of quinoa depending on different screw speeds of extruder.

Sample	Corn Flour (%)	Quinoa Flour (%)	Screw Speed (rpm)
1	100	0	350
2	100	0	500
3	100	0	650
4	90	10	350
5	90	10	500
6	90	10	650
7	80	20	350
8	80	20	500
9	80	20	650
10	70	30	350
11	70	30	500
12	70	30	650

**Table 3:** Physical and technological parameters of extruded products obtained at varying quinoa contents and screw speeds.

Sample	Screw Speed (rpm)	BD (kg/m^3^)	EI	Har (N)	NF	Crw (Nmm)
Extruded products with 0% quinoa fortification						
1	350	21.11 ± 0.46 ^g^	2.22 ± 0.04 ^a^	85.63 ± 7.01 ^e^	11.4 ± 0.86 ^a^	11.74 ± 1.44 ^f^
2	500	15.36 ± 0.32 ^f^	2.51 ± 0.07 ^b–d^	66.23 ± 1.45 ^d^	20.17 ± 0.79 ^bc^	6.49 ± 0.55 ^e^
3	650	9.24 ± 0.25 ^c^	2.71 ± 0.08 ^d^	40.78 ± 1.02 ^ab^	24.5 ± 0.25 ^cd^	5.76 ± 0.82 ^de^
Extruded products with 10% quinoa fortification						
4	350	16.26 ± 0.23 ^f^	2.39 ± 0.07 ^ab^	64.32 ± 5.62 ^cd^	16.5 ± 1.13 ^ab^	4.95 ± 0.82 ^c–e^
5	500	12.78 ± 0.31 ^e^	2.62 ± 0.14 ^b–d^	50.58 ± 8.88 ^bc^	31.6 ± 3.38 ^ef^	3.65 ± 0.70 ^a–d^
6	650	7.73 ± 0.30 ^b^	3.14 ± 0.12 ^e^	35.32 ± 4.38 ^ab^	37.14 ± 0.42 ^fg^	2.79 ± 067 ^ab^
Extruded products with 20% quinoa fortification						
7	350	15.84 ± 0.83 ^f^	2.40 ± 0.09 ^a–c^	59.74 ± 5.35 ^cd^	21.33 ± 1.36 ^b–d^	4.72 ± 0.24 ^b–e^
8	500	10.78 ± 0.31 ^d^	2.68 ± 0.07 ^cd^	42.7 ± 2.62 ^ab^	32.83 ± 1.81 ^ef^	3.45 ± 0.64 ^a–c^
9	650	7.72 ± 0.16 ^b^	3.1 ± 0.13 ^e^	32.06 ± 6.92 ^a^	47.67 ± 0.83 ^h^	2.47 ± 0.29 ^s^
Extruded products with 30% quinoa fortification						
10	350	13.9 ± 0.40 ^e^	2.59 ± 0.10 ^b–d^	43.29 ± 1.15 ^ab^	28.17 ± 2.64 ^de^	4.24 ± 0.59 ^a–d^
11	500	9.28 ± 0.2 ^c^	2.76 ± 0.11 ^d^	33.3 ± 7.55 ^a^	34.67 ± 4.82 ^ef^	3.03 ± 0.71 ^a–c^
12	650	6.25 ± 0.35 ^a^	3.25 ± 0.10 ^e^	28.49 ± 3.11 ^a^	43.83 ± 4.02 ^gh^	2.22 ± 0.38 ^s^

BD—bulk density, EI—expansion index, Har—hardness, NF—number of fractures, Crw—crispiness work (Nmm). Results represent average value (*n* = 6) ± standard deviation. Different letters in superscript of the same table column indicate the statistically significant difference between values, at a level of significance of *p* < 0.05 (based on post hoc Tukey’s HSD test).

**Table 4:** Extruded products’ instrumental color characteristics, depending on different quinoa quantity additions and extruder screw speeds.

Sample	Screw Speed (rpm)	L*	a*	b*	ΔE
Extruded products with 0% quinoa fortification					
1	350	87.32 ± 0.31 ^ef^	0.49 ± 0.31 ^f^	36.09 ± 0.31 ^e^	−
2	500	87.76 ± 0.30 ^fg^	0.32 ± 0.14 ^f^	36.10 ± 0.27 ^e^	0.47 ± 0.07 ^a^
3	650	88.20 ± 0.06 ^gh^	0.19 ± 0.14 ^f^	36.72 ± 0.27 ^e^	1.12 ± 0.09 ^a^
Extruded products with 10% quinoa fortification					
4	350	86.20 ± 0.58 ^de^	−0.41 ± 0.11 ^e^	33.26 ± 2.38 ^cd^	3.17 ± 0.52 ^ab^
5	500	86.87 ± 0.17 ^ef^	−0.70 ± 0.08 ^b−e^	33.72 ± 0.30 ^d^	2.68 ± 0.28 ^ab^
6	650	88.55 ± 0.18 ^h^	−0.98 ± 0.06 ^a−c^	33.28 ± 0.45 ^cd^	3.40 ± 0.44 ^ab^
Extruded products with 20% quinoa fortification					
7	350	85.09 ± 0.28 ^a−c^	−0.51 ± 0.13 ^de^	31.93 ± 0.36 ^b−d^	4.82 ± 0.34 ^ab^
8	500	85.74 ± 0.12 ^b−d^	−0.80 ± 0.05 ^a−f^	31.65 ± 0.39 ^a−d^	4.88 ± 0.45 ^ab^
9	650	86.37 ± 0.22 ^de^	−0.86 ± 0.12 ^a−d^	31.41 ± 0.19 ^a−c^	4.96 ± 0.34 ^ab^
Extruded products with 30% quinoa fortification					
10	350	84.55 ± 0.05 ^a^	−0.67 ± 0.06 ^c−e^	29.77 ± 0.53 ^ab^	6.99 ± 0.36 ^ab^
11	500	84.88 ± 0.12 ^ab^	−1.14 ± 0.27 ^ab^	29.64 ± 0.26 ^a^	7.08 ± 0.13 ^ab^
12	650	85.80 ± 0.24 ^cd^	−1.22 ± 0.10 ^a^	30.18 ± 0.45 ^ab^	6.33 ± 0.42 ^b^

L—brightness (black is 0/white is 100); a—share of red (+)/green tone (−); b—share of yellow (+)/blue tone; ΔΕ—color variation. Results represent average value (*n* = 6) ± standard deviation. Different letters in superscript of the same table column indicate the statistically significant difference between values, at a level of significance of *p* < 0.05 (based on post hoc Tukey’s HSD test).

**Table 5:** Extruded products’ chemical composition, depending on different quinoa quantity additions and extruder screw speeds.

Sample	Screw Speed (rpm)	Moisture (%)	Proteins (% d.m.)	Starch (% d.m.)	Total Sugars (% d.m.)	Total Carbohydrates (% d.m)	Lipids (% d.m.)	Cellulose (% d.m.)	Ash (% d.m.)	Total Dietary Fiber (g/100 g)
Extruded products with 0% quinoa fortification										
1	350	9.54 ± 0.04 ^d^	7.50 ± 0.02 ^a^	82.36 ± 0.70 ^d^	1.83 ± 0.03 ^e^	83.03 ± 0.80 ^bc^	1.12 ± 0.02 ^a^	1.97 ± 0.01 ^a^	0.28 ± 0.00 ^a^	5.70 ± 0.05 ^bc^
2	500	9.22 ± 0.11 ^bc^	7.45 ± 0.03 ^a^	79.07 ± 0.06 ^c^	1.82 ± 0.02 ^e^	83.59 ± 0.12 ^c^	1.12 ± 0.01 ^a^	2.14 ± 0.02 ^b^	0.27 ± 0.01 ^a^	5.62 ± 0.08 ^a–c^
3	650	9.14 ± 0.06 ^bc^	7.35 ± 0.08 ^a^	74.06 ± 0.23 ^a^	1.75 ± 0.03 ^b–d^	83.34 ± 0.80 ^c^	1.13 ± 0.01 ^a^	2.14 ± 0.02 ^b^	0.28 ± 0.00 ^a^	5.48 ± 0.04 ^a^
Extruded products with 10% quinoa fortification										
4	350	9.21 ± 0.08 ^c^	8.42 ± 0.06 ^b^	81.48 ± 0.59 ^d^	1.82 ± 0.01 ^e^	82.60 ± 0.99 ^a–c^	1.36 ± 0.02 ^b^	2.22 ± 0.02 ^cd^	0.50 ± 0.01 ^c^	5.73 ± 0.11 ^c^
5	500	9.09 ± 0.10 ^a–c^	8.62 ± 0.11 ^b^	78.50 ± 0.31 ^c^	1.81 ± 0.02 ^de^	82.37 ± 1.07 ^a–c^	1.40 ± 0.01 ^b^	2.20 ± 0.02 ^bc^	0.49 ± 0.00 ^bc^	5.68 ± 0.04 ^bc^
6	650	8.92 ± 0.13 ^ab^	8.34 ± 0.17 ^b^	76.01 ± 0.71 ^ab^	1.71 ± 0.01 ^ab^	82.28 ± 0.86 ^a–c^	1.38 ± 0.01 ^b^	2.23 ± 0.02 ^cd^	0.48 ± 0.01 ^b^	5.53 ± 0.04 ^ab^
Extruded products with 20% quinoa fortification										
7	350	9.11 ± 0.11 ^c^	9.26 ± 0.12 ^de^	79.05 ± 0.64 ^c^	1.82 ± 0.01 ^e^	81.72 ± 0.45 ^a–c^	1.66 ± 0.02 ^c^	2.25 ± 0.04 ^cd^	0.56 ± 0.00 ^e^	6.20 ± 0.11 ^de^
8	500	9.08 ± 0.09 ^a–c^	8.96 ± 0.04 ^cd^	77.52 ± 0.63 ^bc^	1.80 ± 0.02 ^c–e^	81.45 ± 0.75 ^a–c^	1.63 ± 0.03 ^c^	2.28 ± 0.03 ^de^	0.57 ± 0.01 ^e^	6.18 ± 0.06 ^de^
9	650	8.93 ± 0.06 ^a^	8.88 ± 0.06 ^c^	75.50 ± 0.67 ^a^	1.70 ± 0.02 ^ab^	81.32 ± 0.61 ^a–c^	1.64 ± 0.02 ^c^	2.25 ± 0.03 ^cd^	0.54 ± 0.01 ^d^	6.15 ± 0.05 ^d^
Extruded products with 30% quinoa fortification										
10	350	9.00 ± 0.13 ^a–c^	9.85 ± 0.19 ^g^	77.92 ± 0.46 ^bc^	1.78 ± 0.01 ^c–e^	80.40 ± 1.15 ^a^	2.25 ± 0.03 ^d^	2.34 ± 0.02 ^ef^	0.65 ± 0.01 ^f^	6.35 ± 0.08 ^e^
11	500	8.94 ± 0.17 ^ab^	9.49 ± 0.07 ^f^	76.54 ± 0.87 ^ab^	1.74 ± 0.01 ^a–c^	80.54 ± 1.47 ^ab^	2.35 ± 0.04 ^e^	2.36 ± 0.03 ^f^	0.70 ± 0.01 ^h^	6.32 ± 0.06 ^de^
12	650	8.95 ± 0.13 ^a–c^	9.35 ± 0.11 ^ef^	73.82 ± 1.03 ^a^	1.68 ± 0.02 ^a^	80.58 ± 0.80 ^ab^	2.29 ± 0.05 ^de^	2.35 ± 0.03 ^ef^	0.68 ± 0.01 ^g^	6.26 ± 0.08 ^de^

Results represent average value (*n* = 6) ± standard deviation. Different letters in superscript of the same table column indicate the statistically significant difference between values, at a level of significance of *p* < 0.05 (based on post hoc Tukey’s HSD test.

**Table 6:** Extruded products’ mineral matter composition, depending on different quinoa quantity additions and extruder screw speeds.

Sample	Screw Speed (rpm)	Zn (mg/kg)	Cu (mg/kg)	Fe (mg/kg)	K (mg/kg)	Mg (mg/kg)	Ca (mg/kg)	Mn (mg/kg)	Na (mg/kg)
Extruded products with 0% quinoa fortification									
1	350	4.30 ± 0.03 ^b^	<1.25 ± 0.00 ^a^	6.21 ± 0.07 ^a^	968.88 ± 8.58 ^b^	220.31 ± 1.87 ^a^	26.01 ± 0.33 ^a^	1.24 ± 0.01 ^b^	170.68 ± 1.14 ^f^
2	500	4.02 ± 0.06 ^a^	<1.25 ± 0.00 ^a^	6.06 ± 0.03 ^a^	898.10 ± 0.34 ^a^	229.30 ± 1.61 ^a^	25.85 ± 0.02 ^a^	1.13 ± 0.01 ^a^	183.51 ± 0.19 ^g^
3	650	4.22 ± 0.04 ^b^	<1.25 ± 0.00 ^a^	6.77 ± 0.09 ^b^	945.06 ± 11.75 ^ab^	219.22 ± 0.74 ^a^	24.98 ± 0.27 ^a^	1.22 ± 0.01 ^ab^	168.62 ± 1.86 ^f^
Extruded products with 10% quinoa fortification									
4	350	5.72 ± 0.13 ^c^	1.31 ± 0.01 ^b^	9.62 ± 0.07 ^d^	1434.98 ± 14.84 ^c^	369.63 ± 3.47 ^c^	34.54 ± 0.20 ^bc^	2.21 ± 0.03 ^c^	150.69 ± 0.62 ^e^
5	500	6.01 ± 0.11 ^d^	<1.25 ± 0.00 ^a^	9.05 ± 0.10 ^c^	1405.35 ± 10.73 ^c^	357.33 ± 5.53 ^b^	34.05 ± 0.49 ^b^	2.29 ± 0.02 ^cd^	145.14 ± 0.89 ^d^
6	650	6.26 ± 0.05 ^e^	1.34 ± 0.02 ^b^	9.86 ± 0.09 ^d^	1416.53 ± 11.11 ^c^	387.76 ± 1.31 ^d^	35.21 ± 0.24 ^c^	2.36 ± 0.02 ^d^	138.65 ± 1.53 ^c^
Extruded products with 20% quinoa fortification									
7	350	7.50 ± 0.02 ^f^	1.63 ± 0.01 ^e^	14.27 ± 0.07 ^ef^	1717.12 ± 24.11 ^d^	446.06 ± 4.78 ^f^	41.77 ± 0.64 ^d^	3.36 ± 0.02 ^e^	126.67 ± 1.26 ^b^
8	500	7.72 ± 0.09 ^g^	1.55 ± 0.03 ^d^	13.84 ± 0.08 ^e^	1888.34 ± 21.66 ^e^	441.82 ± 4.84 ^f^	42.17 ± 0.49 ^d^	3.47 ± 0.06 ^f^	137.50 ± 1.32 ^c^
9	650	7.85 ± 0.13 ^g^	1.46 ± 0.01 ^c^	14.55 ± 0.05 ^f^	1899.84 ± 30.20 ^e^	427.68 ± 6.81 ^e^	42.85 ± 0.43 ^d^	3.75 ± 0.01 ^g^	130.87 ± 1.34 ^b^
Extruded products with 30% quinoa fortification									
10	350	9.87 ± 0.10 ^j^	1.75 ± 0.00 ^fg^	17.47 ± 0.06 ^g^	2177.22 ± 15.10 ^g^	562.02 ± 6.94 ^i^	50.54 ± 0.35 ^e^	4.57 ± 0.07 ^i^	85.65 ± 1.35 ^a^
11	500	9.26 ± 0.07 ^h^	1.72 ± 0.03 ^f^	17.61 ± 0.19 ^g^	2225.50 ± 21.90 ^f^	513.27 ± 2.77 ^g^	51.14 ± 0.62 ^e^	4.36 ± 0.00 ^h^	90.21 ± 1.08 ^a^
12	650	9.57 ± 0.11 ^i^	1.78 ± 0.02 ^g^	18.20 ± 0.31 ^h^	2061.55 ± 29.62 ^g^	529.00 ± 5.96 ^h^	51.64 ± 0.60 ^e^	4.32 ± 0.02 ^h^	86.79 ± 0.38 ^a^

Results represent average value (*n* = 6) ± standard deviation. Different letters in superscript of the same table column indicate the statistically significant difference between values, at a level of significance of *p* < 0.05 (based on post hoc Tukey’s HSD test). The reported Cu content of “<1.25 ± 0.00 mg/kg” corresponds to the detection limit; values below this limit cannot be quantified accurately, and therefore are reported as less than the detection threshold.

**Table 7:** Extruded products’ essential amino acids’ content, depending on different quinoa quantity additions and extruder screw speeds.

Sample	Screw Speed (rpm)	Isoleucine (g/100 g of Protein)	Leucine (g/100 g of Protein)	Lysine (g/100 g of Protein)	Methionine + Cystine (g/100 g of Protein)	Phenylalanine + Tyrosine (g/100 g of Protein)	Threonine (g/100 g of Protein)	Tryptophan (g/100 g of Protein)	Valine (g/100 g of Protein)	Total Essential Amino Acids (g/100 g of Protein)
Extruded products with 0% quinoa fortification										
1	350	3.30 ± 0.01 ^a^	11.83 ± 0.10 ^d–f^	2.21 ± 0.02 ^a^	3.10 ± 0.03 ^ab^	8.24 ± 0.09 ^ab^	3.59 ± 0.04 ^a^	0.70 ± 0.01 ^a^	4.65 ± 0.04 ^a^	37.81 ± 0.41 ^a–c^
2	500	3.41 ± 0.03 ^ab^	12.05 ± 0.09 ^fg^	2.47 ± 0.03 ^b^	3.33 ± 0.06 ^c^	8.46 ± 0.08 ^b–d^	3.69 ± 0.03 ^a–d^	0.75 ± 0.01 ^bc^	4.73 ± 0.03 ^a–c^	39.10 ± 0.44 ^de^
3	650	3.46 ± 0.02 ^bc^	12.32 ± 0.13 ^g^	2.57 ± 0.03 ^c^	3.49 ± 0.03 ^d^	8.85 ± 0.12 ^e^	3.74 ± 0.04 ^b–d^	0.77 ± 0.00 ^cd^	4.81 ± 0.03 ^c–e^	40.01 ± 0.40 ^e^
Extruded products with 10% quinoa fortification										
4	350	3.39 ± 0.04 ^ab^	11.42 ± 0.14 ^c^	2.41 ± 0.01 ^b^	3.11 ± 0.04 ^ab^	8.22 ± 0.15 ^ab^	3.61 ± 0.02 ^ab^	0.73 ± 0.01 ^b^	4.67 ± 0.03 ^ab^	37.19 ± 0.28 ^ab^
5	500	3.46 ± 0.02 ^bc^	11.63 ± 0.13 ^c–e^	2.61 ± 0.02 ^c^	3.21 ± 0.03 ^b^	8.43 ± 0.03 ^b–d^	3.69 ± 0.07 ^a–d^	0.78 ± 0.00 ^d^	4.75 ± 0.05 ^a–c^	38.91 ± 0.29 ^c–e^
6	650	3.55 ± 0.02 ^c–e^	11.88 ± 0.12 ^ef^	2.89 ± 0.02 ^e^	3.42 ± 0.05 ^cd^	8.77 ± 0.11 ^e^	3.79 ± 0.04 ^c–e^	0.81 ± 0.01 ^ef^	4.82 ± 0.05 ^c–e^	39.19 ± 0.37 ^de^
Extruded products with 20% quinoa fortification										
7	350	3.49 ± 0.05 ^b–d^	10.84 ± 0.15 ^b^	2.60 ± 0.01 ^c^	3.04 ± 0.03 ^a^	8.18 ± 0.11 ^ab^	3.68 ± 0.02 ^a–c^	0.79 ± 0.00 ^de^	4.65 ± 0.09 ^a^	37.04 ± 0.42 ^ab^
8	500	3.58 ± 0.02 ^de^	11.37 ± 0.05 ^c^	2.81 ± 0.02 ^d^	3.20 ± 0.03 ^b^	8.37 ± 0.07 ^a–c^	3.75 ± 0.06 ^cd^	0.82 ± 0.01 ^f^	4.77 ± 0.05 ^a–e^	38.49 ± 0.54 ^cd^
9	650	3.61 ± 0.04 ^e^	11.49 ± 0.03 ^cd^	3.09 ± 0.03 ^f^	3.41 ± 0.02 ^cd^	8.70 ± 0.12 ^de^	3.82 ± 0.03 ^de^	0.86 ± 0.01 ^g^	4.87 ± 0.01 ^de^	38.99 ± 0.59 ^c–e^
Extruded products with 30% quinoa fortification										
10	350	3.58 ± 0.03 ^de^	10.41 ± 0.11 ^a^	2.75 ± 0.04 ^d^	3.00 ± 0.03 ^a^	8.10 ± 0.15 ^a^	3.72 ± 0.02 ^a–d^	0.82 ± 0.02 ^f^	4.68 ± 0.04 ^sb^	36.81 ± 0.41 ^a^
11	500	3.62 ± 0.03 ^e^	10.89 ± 0.10 ^b^	3.18 ± 0.05 ^g^	3.11 ± 0.01 ^ab^	8.30 ± 0.09 ^ab^	3.79 ± 0.05 ^c–e^	0.86 ± 0.01 ^g^	4.79 ± 0.08 ^b–e^	38.07 ± 0.67 ^b–d^
12	650	3.75 ± 0.04 ^f^	10.99 ± 0.09 ^b^	3.38 ± 0.03 ^h^	3.37 ± 0.05 ^c^	8.67 ± 0.09 ^c–e^	3.89 ± 0.05 ^e^	0.91 ± 0.01 ^h^	4.88 ± 0.04 ^e^	38.51 ± 0.42 ^cd^

Results represent average value (*n* = 6) ± standard deviation. Different letters in superscript of the same table column indicate the statistically significant difference between values, at a level of significance of *p* < 0.05 (based on post hoc Tukey’s HSD test).

**Table 8:** Fatty acid composition of extruded products as affected by quinoa addition level and extrusion screw speed.

Sample	Screw Speed (rpm)	Palmitic Acid (g/100 g of Fat)	Stearic Acid (g/100 g of Fat)	Oleic Acid (g/100 g of Fat)	Linoleic Acid (g/100 g of Fat)	Linolenic Acid (g/100 g of fat)	Total Fatty Acids (g/100 g of Fat)
Extruded products with 0% quinoa fortification							
1	350	10.61 ± 0.12 ^a^	2.40 ± 0.03 ^a^	26.41 ± 0.33 ^c^	51.03 ± 0.41 ^de^	0.58 ± 0.01 ^a^	91.19 ± 1.38 ^a^
2	500	10.90 ± 0.08 ^ab^	2.41 ± 0.02 ^a^	27.29 ± 0.35 ^d^	53.18 ± 0.47 ^fg^	0.55 ± 0.00 ^a^	94.07 ± 0.68 ^a–d^
3	650	10.95 ± 0.12 ^a–c^	2.49 ± 0.01 ^a^	27.68 ± 0.21 ^d^	54.91 ± 0.51 ^g^	0.64 ± 0.01 ^b^	96.99 ± 0.69 ^de^
Extruded products with 10% quinoa fortification							
4	350	11.15 ± 0.07 ^b–d^	2.62 ± 0.05 ^bc^	26.01 ± 0.37 ^bc^	49.46 ± 0.24 ^cd^	1.00 ± 0.01 ^c^	92.59 ± 0.33 ^a–c^
5	500	11.30 ± 0.05 ^c–e^	2.60 ± 0.02 ^b^	26.11 ± 0.13 ^bc^	51.08 ± 0.24 ^de^	1.08 ± 0.02 ^d^	94.21 ± 1.21 ^b–e^
6	650	11.49 ± 0.18 ^de^	2.65 ± 0.02 ^bc^	26.43 ± 0.41 ^c^	52.65 ± 0.33 ^ef^	1.18 ± 0.02 ^e^	97.73 ± 0.59 ^f^
Extruded products with 20% quinoa fortification							
7	350	11.68 ± 0.11 ^ef^	2.71 ± 0.04 ^cd^	25.72 ± 0.18 ^a–c^	46.75 ± 0.76 ^b^	1.50 ± 0.01 ^f^	92.99 ± 0.87 ^a–c^
8	500	11.98 ± 0.04 ^fg^	2.80 ± 0.03 ^de^	26.00 ± 0.33 ^bc^	48.06 ± 0.39 ^bc^	1.55 ± 0.02 ^g^	95.19 ± 0.88 ^c–f^
9	650	11.99 ± 0.15 ^fg^	2.85 ± 0.01 ^e^	26.13 ± 0.29 ^bc^	50.87 ± 0.50 ^de^	1.61 ± 0.01 ^h^	97.09 ± 0.99 ^ef^
Extruded products with 30% quinoa fortification							
10	350	12.20 ± 0.05 ^gh^	2.99 ± 0.03 ^f^	25.07 ± 0.17 ^a^	44.28 ± 0.29 ^a^	2.00 ± 0.01 ^i^	92.09 ± 0.75 ^ab^
11	500	12.43 ± 0.25 ^h^	3.05 ± 0.00 ^fg^	25.17 ± 0.29 ^a^	46.44 ± 0.45 ^b^	2.01 ± 0.02 ^i^	94.41 ± 0.82 ^b–e^
12	650	12.48 ± 0.06 ^h^	3.10 ± 0.01 ^g^	25.43 ± 0.27 ^ab^	47.99 ± 0.47 ^bc^	2.13 ± 0.03 ^j^	98.19 ± 0.48 ^f^

Results represent average value (*n* = 6) ± standard deviation. Different letters in superscript of the same table column indicate the statistically significant difference between values, at a level of significance of *p* < 0.05 (based on post hoc Tukey’s HSD test).

## Data Availability

The original contributions presented in the study are included in the article/Supplementary Materials, and further inquiries can be directed to the corresponding author.

## Supplementary Materials

The following supporting information can be downloaded at: https://www.mdpi.com/article/10.3390/foods15020208/s1, Table S1: extruded products’ descriptive sensory analysis form; Table S2: descriptive sensory attributes intensity scale instructions; Table S3: extruded products’ descriptive sensory analysis depending on different quinoa quantity addition and extruder screw speed; Table S4: ANOVA of extruded products with the addition of quinoa quality parameters response models, Table S5: Second order polynom regression coefficients for flips products with the addition of quinoa quality parameters response models, Table S6: Z-Score analysis of extruded product depending on different quinoa quantity addition and extruder screw speed, Figure S1: Graphical presentation of mathematically modeled dependance of extruded products physical and technological characteristics from the share of quinoa and screw speed, (a) Bulk density, (b) Expansion index, (c) Hardness, (d) Number of Fracurability and (e) Crispier work, Figure S2: Graphical presentation of mathematically modeled dependance of extruded products instrumental colour characteristics from the share of quinoa and screw speed, (a) L*, (b) a*, (c) b* and (d) ΔE, Figure S3: Graphical presentation of mathematically modeled dependance of extruded products chemical content from the share of quinoa and screw speed, (a) Moisture, (b) Proteins, (c) Starch, (d) Total sugars, (e) Total carbohydrates, (f) Lipids, (g) Cellulose, (h) Ash and (i) Total dietary fiber, Figure S4: Graphical presentation of mathematically modeled dependance of extruded products mineral matter content from the share of quinoa and screw speed, (a) Zn, (b) Cu, (c) Fe, (d) K, (e) Mg, (f) Ca, (g) Mg and (h) Na, Figure S5: Graphical presentation of mathematically modeled dependance of extruded products essential aminoacids’ content from the share of quinoa and screw speed, (a) Isoleucine, (b) Leucine, (c) Lysine, (d) Metionine + Cystine, (e) Phenylalanine + Tyrosine, (f) Threonine, (g) Tryptophan, (h) Valine and (i) Total essental amino acids, Figure S6: Graphical presentation of mathematically modeled dependance of extruded products non-essential aminoacids’ content from the share of quinoa and screw speed, (a) Alanine, (b) Arginine, (c) Aspartic acid, (d) Glutamic acid, (e) Glycine, (f) Threonine, (g) Proline, (h) Serine and (i) Total non-essental amino acids, Figure S7: Graphical presentation of mathematically modeled dependance of extruded products fatty acids content from the share of quinoa and screw speed, (a) Palmitic fatty acid, (b) Stearic fatty acid, (c) Oleic fatty acid, (d) Linoleic fatty acid, (e) Linolenic fatty acid and (f) Total fatty acids, Figure S8: Graphical presentation of mathematically modeled dependance of extruded products descriptive sensory analysis from the share of quinoa and screw speed, (a) Colour, (b) Shape, (c) Hardness, (d) Crispiness, (e) Expansion perception and (f) Taste.

## Abbreviations

The following abbreviations are used in this manuscript:

RSM	Response surface methodology
BD	Bulk density
EI	Expansion index
Har	Hardness
NF	Number of fractures
Crw	Crispier work
