# Supplementary material for: Modeling and Optimization of Extruded Corn Product Fortification
Source: Foods. 2026 Jan 7;15(2):208. doi: 10.3390/foods15020208 (PMC12840189; doi:10.3390/foods15020208)
Supplement: Supplementary file 1 [file foods-15-00208-s001.zip › foods-4089177-supplementary.pdf]

Modelling and Optimization of Extruded Corn Product Fortification

Jelena Filipović[1], Ivica Djalović[2], Milenko Košutić[1], Milica Nićetin[3*], Biljana Lončar[3], Miloš Radosavljević[3], and Vladimir Filipović[3]

[1] Institute of Food Technology, University of Novi Sad, Bulevar Cara Lazara 1, 21000 Novi Sad, Serbia; milenko.kosutic@fins.uns.ac.rs (M.K); jelena.filipovic@fins.uns.ac.rs (J.F);
[2] Institute of Field and Vegetable Crops, National Institute of the Republic of Serbia, Maxim Gorki 30, 21000 Novi Sad, Serbia ivica.djalovic@ifvcns.ns.ac.rs (I.Dj),
[3] Faculty of Technology Novi Sad, University of Novi Sad, Bulevar Cara Lazara 1, 21000 Novi Sad, Serbia,
vladaf@uns.ac.rs (V.F.); cbiljana@uns.ac.rs (B.L.); milican@uns.ac.rs (M.N.); Milos1506@gmail.com (M.R.)

* Correspondence: milican@uns.ac.rs (M.N.);

Table S1. Extruded products' descriptive sensory analysis form

**Sample no:**									
**Attribute**	9-point intensity scale								
**Colour**	1	2	3	4	5	6	7	8	9
**Shape**	1	2	3	4	5	6	7	8	9
**Hardness**	1	2	3	4	5	6	7	8	9
**Crispiness**	1	2	3	4	5	6	7	8	9
**Expansion perception**	1	2	3	4	5	6	7	8	9
**Taste**	1	2	3	4	5	6	7	8	9

Table S2. Descriptive sensory attributes intensity scale instructions:

Colour:

1	2	3	4	5	6	7	8	9
Extremely pale	Very pale	Moderate pale	Modest pale	Optimal	Modest dark	Moderate dark	Very dark	Extremely dark

Shape:

1	2	3	4	5	6	7	8	9

Extremely deformed	Very deformed	Moderate deformed	Modest deformed	Neither deformed or uniformed	Modest uniformed	Moderate uniformed	Very uniformed	Extremely uniformed

Hardness:

1	2	3	4	5	6	7	8	9
Extremely soft	Very soft	Moderate soft	Modest soft	Optimal	Modest hard	Moderate hard	Very hard	Extremely hard

Crispiness:

1	2	3	4	5	6	7	8	9
Extremely tough	Very tough	Moderate tough	Modest tough	Neither tough or crispy	Modest crispy	Moderate crispy	Very crispy	Extremely crispy

Expansion perception:

1	2	3	4	5	6	7	8	9
Extremely compact	Very compact	Moderate compact	Modest compact	Neither compact or porous	Modest porous	Moderate porous	Very porous	Extremely porous

Taste:

1	2	3	4	5	6	7	8	9

Table S3. Extruded products' descriptive sensory analysis depending on different quinoa quantity addition and extruder screw speed

Sample	Screw speed (rpm)	Colour	Shape	Hardness	Crispiness	Expansion perception	Taste
Extruded products with 0% quinoa fortification							
1	350	4.50±0.10^{a}	8.10±0.10^{a}	8.50±0.15^{g}	2.75±0.25^{a}	1.75±0.25^{a}	3.90±0.10^{a}
2	500	4.75±0.05^{a}	8.50±0.15^{ab}	7.40±0.10^{f}	3.33±0.33^{a}	2.00±0.10^{a}	4.50±0.25^{ab}
3	650	5.33±0.13^{b}	8.75±0.25^{b}	5.25±0.25^{bc}	4.33±0.16^{b}	2.33±0.33^{a}	5.16±0.16^{b}
Extruded products with 10% quinoa fortification							
4	350	6.33±0.16^{de}	8.20±0.20^{ab}	6.50±0.10^{e}	5.25±0.25^{c}	1.90±0.10^{a}	6.33±0.33^{cd}
5	500	5.75±0.25^{bc}	8.33±0.33^{ab}	5.75±0.25^{cd}	6.10±0.10^{de}	3.25±0.25^{b}	6.20±0.20^{cd}
6	650	5.40±0.10^{b}	8.75±0.25^{b}	5.16±0.15^{bc}	6.33±0.33^{de}	5.00±0.25^{c}	5.90±0.10^{c}
Extruded products with 20% quinoa fortification							
7	350	7.50±0.25^{fg}	8.16±0.16^{ab}	6.00±0.10^{de}	5.75±0.25^{cd}	3.50±0.25^{b}	6.66±0.16^{de}
8	500	6.50±0.10^{e}	8.50±0.10^{ab}	5.25±0.25^{bc}	6.66±0.33^{ef}	4.75±0.25^{c}	6.50±0.50^{cd}
9	650	6.00±0.10^{cd}	8.60±0.15^{ab}	4.75±0.33^{ab}	7.33±0.33^{f}	6.16±0.16^{d}	6.33±0.33^{cd}
Extruded products with 30% quinoa fortification							

10	350	7.75±0.25^{g}	8.33±0.33^{ab}	4.75±0.25^{ab}	6.33±0.33^{de}	6.50±0.50^{d}	7.50±0.10^{f}
11	500	7.16±0.15^{f}	8.40±0.10^{ab}	4.33±0.10^{a}	6.66±0.16^{ef}	7.33±0.16^{e}	7.33±0.16^{ef}
12	650	6.40±0.10^{de}	8.50±0.25^{ab}	4.33±0.33^{a}	7.16±0.16^{f}	8.16±0.25^{f}	7.25±0.25^{ef}

Results represent average value (n = 6) ± standard deviation. Different letters in superscript of the same table column indicate the statistically significant difference between values, at a level of significance of $p < 0.05$ (based on post-hoc Tukey HSD test)

Table S4. ANOVA of extruded products with the addition of quinoa quality parameters response models

Technological parameters	Share of quinoa		Screw speed		Cross product	Error		R^2
Term	Linear	Quadra-tic	Linear	Quadra-tic	Share of quinoa x screw speed	Residual variance	Total sum of squares	
df+	1	1	1	1	1	6	11	
Physical and technological characteristics								
Bulk density	43.81*	1.35	163.53*	0.11	4.27*	4.01	217.09	0.98
Expansion index	0.21*	0.01	0.85*	0.02	0.01	0.05	1.13	0.96
Hardness	1289.83*	13.93	1693.62*	0.65	210.13*	62.65	3270.81	0.98
Number of Fracurability	472.59	49.33	717.07	2.67	4.48	65.68	1311.81	0.95
Crispier work	32.63*	10.93*	19.25*	1.33	3.48	6.71	74.32	0.91
Instrumental colour characteristics								
L*	8.20*	0.04	3.35*	0.05	0.02	1.13	18.20	0.94
a*	1.60*	0.31	0.26	0.01	0.01	0.28	2.45	0.89
b*	44.22*	0.71	0.02	0.01	0.02	0.81	56.69	0.99
Δ E	40.95	0.17	0.02	0.03	0.29	0.49	49.50	0.99

Chemical composition								
Moisture	0.16*	0.02	0.11*	0.00	0.03*	0.02	0.34	0.94
Proteins	7.27*	0.19*	0.15*	0.00*	0.05	0.09	7.75	0.99
Starch	10.88*	1.53	57.35*	0.39	5.27*	2.32	77.74	0.97
Total sugars	0.01*	0.00	0.02*	0.00*	0.00	0.00	0.03	0.96
Total carbohydrates	13.54*	0.01	0.00	0.02	0.00	0.33	13.91	0.98
Lipids	2.15*	0.12*	0.00	0.00	0.00	0.03	2.29	0.99
Cellulose	0.11*	0.00	0.00	0.00	0.01	0.01	0.13	0.92
Ash	0.24*	0.01	0.00	0.00	0.00	0.01	0.26	0.97
Total dietary fiber	1.06*	0.01	0.04	0.00	0.01	0.12	1.24	0.90
Mineral composition								
Zn	47.81*	0.00	0.03	0.07	0.02	0.33	48.26	0.99
Cu	0.46*	0.02	0.00	0.00	0.00	0.03	0.50	0.95
Fe	227.57*	0.11	0.41	0.61	0.01	1.26	229.96	0.99
K	2482753	19671	77	1897	138	36216	2540752	0.99
Mg	150722.9*	2058.3	147.6	255.1	437.6	2960.5	156581.8	0.98
Ca	1062.02*	0.02	0.41	0.05	1.16	1.81	1065.47	0.99
Mn	17.82*	0.03	0.01	0.01	0.01	0.15	18.02	0.99
Na	11204.48*	161.77	9.59	121.95	16.69	460.78	11975.27	0.96
Essential aminoacids' composition								
Isoleucine	0.11*	0.00	0.05*	0.00	0.00	0.00	0.16	0.98
Leucine	2.80*	0.00	0.59*	0.02	0.01	0.04	3.46	0.99
Lysine	0.76*	0.00	0.48*	0.00	0.02*	0.02	1.28	0.98
Metionine + Cystine	0.03*	0.00	0.26*	0.00	0.00	0.01	0.30	0.97

Phenylalanine + Tyrosine	0.04*	0.00	0.63*	0.02*	0.00	0.00	0.69	0.99
Threonine	0.03*	0.00	0.05*	0.00	0.00	0.00	0.08	0.98
Tryptophan	0.03*	0.00	0.01*	0.00	0.00	0.00	0.04	0.99
Valine	0.00*	0.00	0.07*	0.00	0.00*	0.00	0.07	0.99
Total essental amino acids	2.15*	0.02	7.70*	0.54*	0.06	0.12	10.59	0.98
Non-essential aminoacids' composition								
Alanine	0.37*	0.00	0.11*	0.00*	0.00	0.00	0.48	0.99
Arginine	1.21*	0.00	0.10*	0.00	0.00*	0.00	1.32	0.99
Aspartic acid	0.40*	0.00	0.06*	0.00	0.00	0.00	0.47	0.99
Glutamic acid	2.57*	0.00	0.34*	0.00	0.01	0.01	2.92	0.99
Glycine	0.17*	0.00	0.02*	0.00	0.00	0.00	0.19	0.99
Histidine	0.03*	0.00	0.03*	0.00	0.00	0.00	0.07	0.97
Proline	1.99*	0.00	0.05*	0.00	0.00*	0.00	2.04	0.99
Serine	0.00*	0.00	0.02*	0.00	0.00	0.00	0.02	0.94
Total non-essental amino acids	2.93*	0.05*	7.53*	0.02*	0.00	0.01	10.54	0.99
Fatty acids composition								
Palmitic fatty acid	4.09*	0.00	0.20*	0.02*	0.00	0.01	4.32	0.99
Stearic fatty acid	0.60*	0.00	0.02*	0.00	0.00	0.01	0.63	0.98
Oleic fatty acid	5.30*	0.04	0.76*	0.00	0.19	0.37	6.65	0.94
Linoleic fatty acid	78.75	0.09	27.75	0.01	0.00	0.76	107.37	0.99
Linolenic fatty acid	3.51*	0.00	0.03*	0.00	0.00	0.01	3.55	0.99
Total fatty acids	1.08	0.68	55.86*	0.40	0.00	1.75	59.78	0.97

Descriptive sensory analysis								
Colour	8.60*	0.21	1.09*	0.03	1.26*	0.51	11.70	0.96
Shape	0.00	0.00	0.41*	0.00	0.06*	0.03	0.50	0.94
Hardness	10.11	0.11	4.90	0.00	1.84	0.74	17.70	0.96
Crispiness	16.31*	3.92*	3.21*	0.00	0.08	0.45	23.97	0.98
Expansion perception	45.05*	1.03	8.00*	0.02	0.20	1.87	56.16	0.97
Taste	11.81*	0.43	0.01	0.00	0.49	0.96	13.70	0.93

[+] Degrees of freedom, * Statistically significant at level of $p<0.05$

Table S5. Second order polynom regression coefficients for flips products with the addition of quinoa quality parameters response models

	β_{k0}	β_{k1}	β_{k11}	β_{k2}	β_{k22}	β_{k12}
Physical and technological characteristics						
Bulk density	35.58*	-0.49*	0.00	-0.05	0.00	0.00*
Expansion index	2.39*	0.01	0.00	0.00	0.00	0.00
Hardness	140.08*	-2.78*	0.01	-0.16	0.00	0.00*
Number of Fracurability	-19.95	0.95	-0.02	0.10	0.00	0.00
Crispier work	23.29*	-0.63*	0.01*	-0.05	0.00	0.00
Instrumental colour characteristics						
L*	86.33*	-0.05	-0.00	-0.00	0.00	-0.00
a*	1.65	-0.08	0.00	0.00	0.00	0.00
b*	36.41*	-0.28*	0.00	0.00	0.00	0.00
Δ E	40.95*	0.17	0.02	0.03	0.29	0.49
Chemical composition						
Moisuture	10.14*	-0.04*	0.00	-0.00	0.00	0.00*
Proteins	7.46*	0.13*	0.00*	0.00	0.00	0.00
Starch	87.13*	-0.22*	0.00	-0.01	0.00	0.00*
Total sugars	1.62*	0.00	0.00	0.00*	0.00*	0.00
Total carbohydrates	82.33*	-0.08*	0.00	0.00	0.00	0.00
Lipids	0.91	0.01	0.00*	0.00	0.00	0.00
Cellulose	1.61*	0.02*	0.00	0.00	0.00	0.00
Ash	0.20	0.02*	0.00	0.00	0.00	0.00
Total dietary fiber	5.66*	0.01	0.00	0.00	0.00	0.00
Mineral composition						
Zn	5.43*	0.19*	0.00	-0.01	0.00	0.00

Cu	1.56*	0.01	0.00	0.00	0.00	0.00
Fe	10.59*	0.35*	0.00	-0.02	0.00	0.00
K	628.27	54.07*	-0.40	1.24	0.00	0.00
Mg	311.88	16.16*	-0.13	-0.40	0.00	0.00
Ca	28.14*	0.74*	0.00	-0.01	0.00	0.00
Mn	1.64	0.13*	0.00	0.00	0.00	0.00
Na	111.39	-2.06	-0.04	0.28	0.00	0.00
Essential aminoacids' composition						
Isoleucine	3.12*	0.01	0.00	0.00	0.00	0.00
Leucine	10.36*	-0.05*	0.00	0.01*	0.00	0.00
Lysine	1.50*	0.01	0.00	0.00	0.00	0.00*
Metionine + Cystine	3.02*	0.00	0.00	0.00	0.00	0.00
Phenylalanine + Tyrosine	8.34*	0.00	0.00	0.00*	0.00*	0.00
Threonine	3.41*	0.00	0.00	0.00	0.00	0.00
Tryptophan	0.58*	0.00*	0.00	0.00	0.00	0.00
Valine	4.41*	0.00	0.00	0.00*	0.00	0.00*
Total essental amino acids	30.61*	-0.02	0.00	0.03*	0.00*	0.00
Non-essential aminoacids' composition						
Alanine	9.16*	-0.02*	0.00	0.00	0.00*	0.00
Arginine	3.50*	0.02*	0.00	0.00*	0.00	0.00*
Aspartic acid	8.03*	0.01*	0.00	0.00	0.00	0.00
Glutamic acid	19.06*	-0.03*	0.00	0.00	0.00	0.00
Glycine	3.55*	0.01*	0.00	0.00	0.00	0.00
Histidine	2.50*	0.01*	0.00	0.00	0.00	0.00
Proline	8.33*	-0.04*	0.00	0.00	0.00	0.00*
Serine	4.43*	0.00	0.00	0.00	0.00	0.00

Total non-essental amino acids	54.49*	-0.03*	0.00*	0.01*	0.00*	0.00
Fatty acids composition						
Palmitic fatty acid	9.36*	0.06*	0.00	0.00*	0.00*	0.00
Stearic fatty acid	2.45*	0.01*	0.00	0.00	0.00	0.00
Oleic fatty acid	25.02*	-0.03	0.00	0.00	0.00	0.00
Linoleic fatty acid	47.52*	-0.21*	0.00	0.01	0.00	0.00
Linolenic fatty acid	0.77*	0.05*	0.00	0.00	0.00	0.00
Total fatty acids	89.29*	0.10	0.00	0.00	0.00	0.00
Descriptive sensory analysis						
Colour	5.46*	0.23*	0.00	0.00	0.00	0.00*
Shape	7.22*	0.02	0.00	0.00	0.00	0.00*
Hardness	11.46*	-0.25*	0.00	-0.01	0.00	0.00*
Crispiness	0.63	0.30*	-0.01*	0.01	0.00	0.00
Expansion perception	0.28	0.04	0.00	0.00	0.00	0.00
Taste	3.36	0.22*	0.00	0.00	0.00	0.00

* Statistically significant at level of $p<0.05$

Table S6. Z-Score analysis of extruded product depending on different quinoa quantity addition and extruder screw speed

Sample	Screw speed (rpm)	S1	S2	S3	S4	S5	S6	S7	S8	Total S
Extruded products with 0% quinoa fortification										
1	350	0.20	**0.90**	0.46	0.04	0.16	0.31	**0.69**	0.25	0.34
2	500	0.42	0.89	0.52	0.01	0.46	0.45	0.68	0.49	0.48
3	650	0.50	0.89	0.39	0.04	0.69	0.66	0.65	0.73	0.55
Extruded products with 10% quinoa fortification										
4	350	0.39	0.49	0.64	0.32	0.19	0.30	0.55	0.40	0.42
5	500	0.55	0.52	0.60	0.31	0.45	0.48	0.55	0.58	0.52
6	650	0.71	0.54	0.50	0.37	0.70	0.66	0.49	**0.82**	0.62
Extruded products with 20% quinoa fortification										
7	350	0.42	0.30	**0.69**	0.64	0.22	0.34	0.47	0.37	0.44
8	500	0.57	0.28	0.65	0.63	0.51	0.50	0.41	0.64	0.55
9	650	0.76	0.30	0.55	0.64	0.76	0.69	0.41	0.77	**0.64**
Extruded products with 30% quinoa fortification										
10	350	0.47	0.09	0.66	**0.98**	0.25	0.34	0.35	0.47	0.47
11	500	0.59	0.03	0.62	0.93	0.52	0.51	0.30	0.56	0.54
12	650	**0.78**	0.12	0.52	0.96	**0.82**	**0.69**	0.25	0.68	0.63

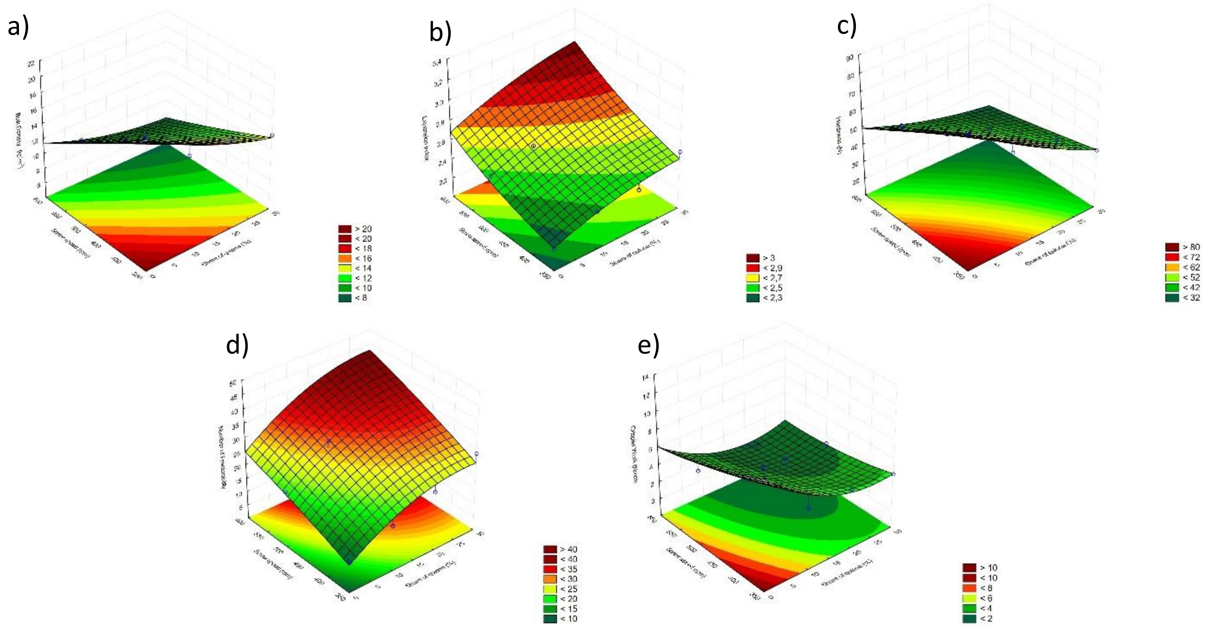

Figure S1. Graphical presentation of mathematically modeled dependance of extruded products physical and technological characteristics from the share of quinoa and screw speed, a) Bulk density, b) Expansion index, c) Hardness, d) Number of Fracurability and e) Crispier work

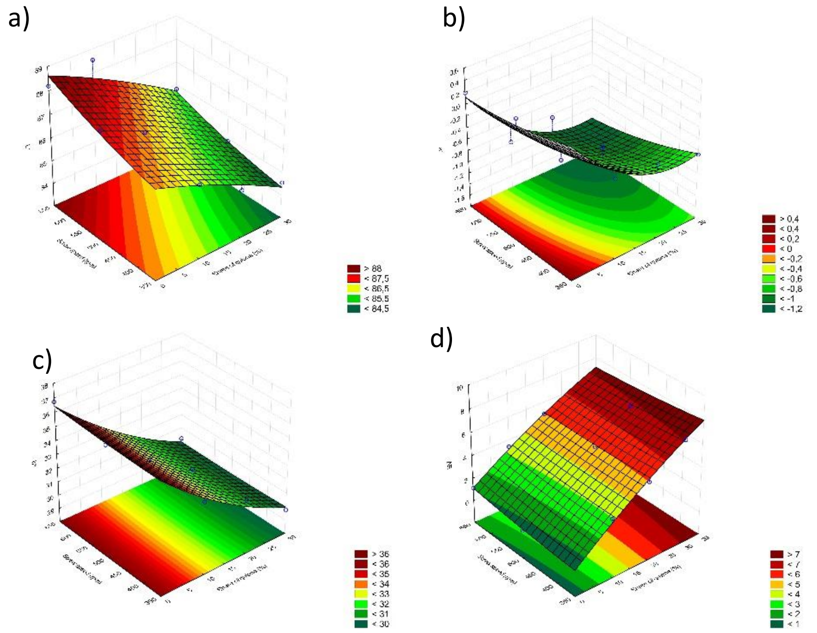

Figure S2. Graphical presentation of mathematically modeled dependance of extruded products instrumental colour characteristics from the share of quinoa and screw speed, a) L*, b) a*, c) b* and d) ΔE

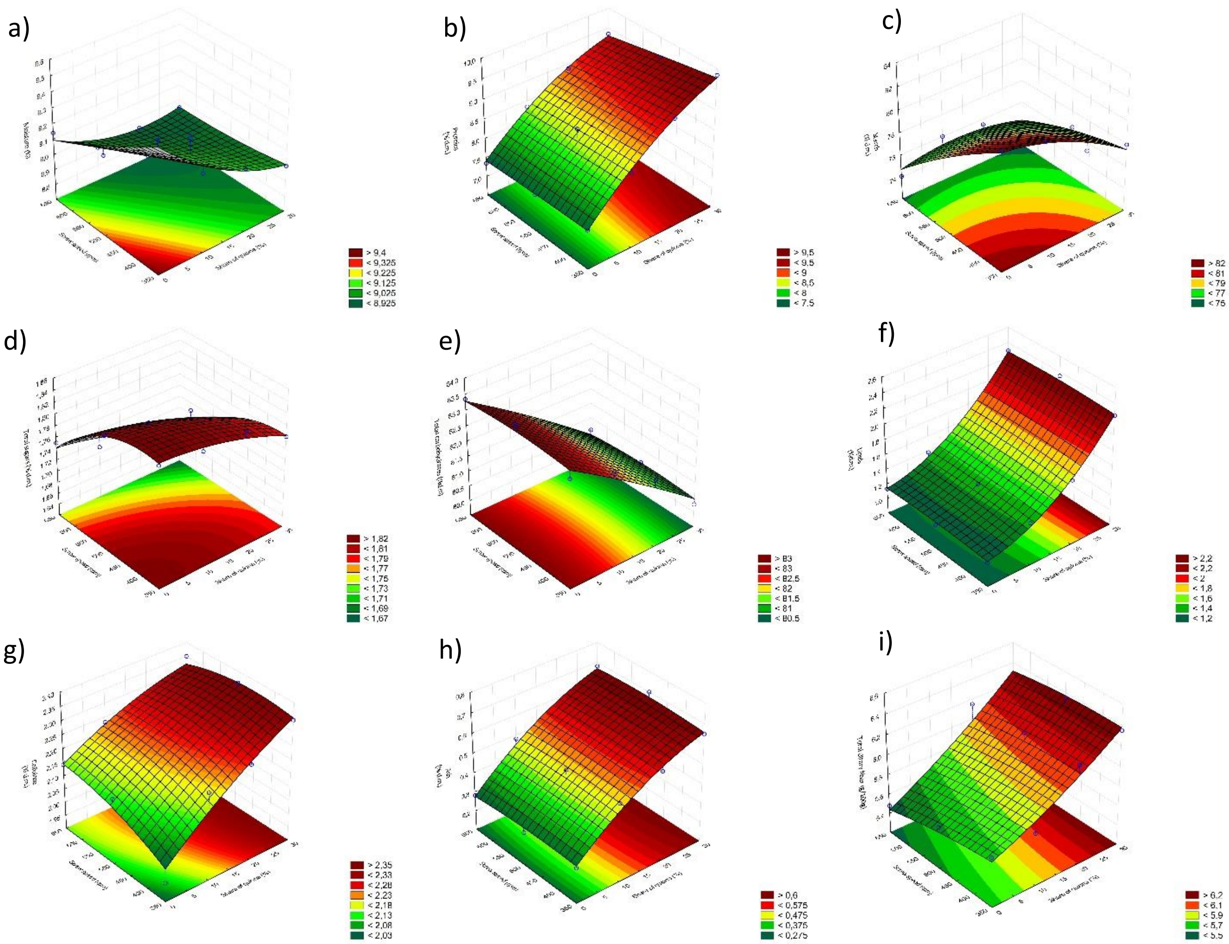

Figure S3. Graphical presentation of mathematically modeled dependance of extruded products chemical content from the share of quinoa and screw speed, a) Moisture, b) Proteins, c) Starch, d) Total sugars, e) Total carbohydrates, f) Lipids, g) Cellulose, h) Ash and i) Total dietary fiber

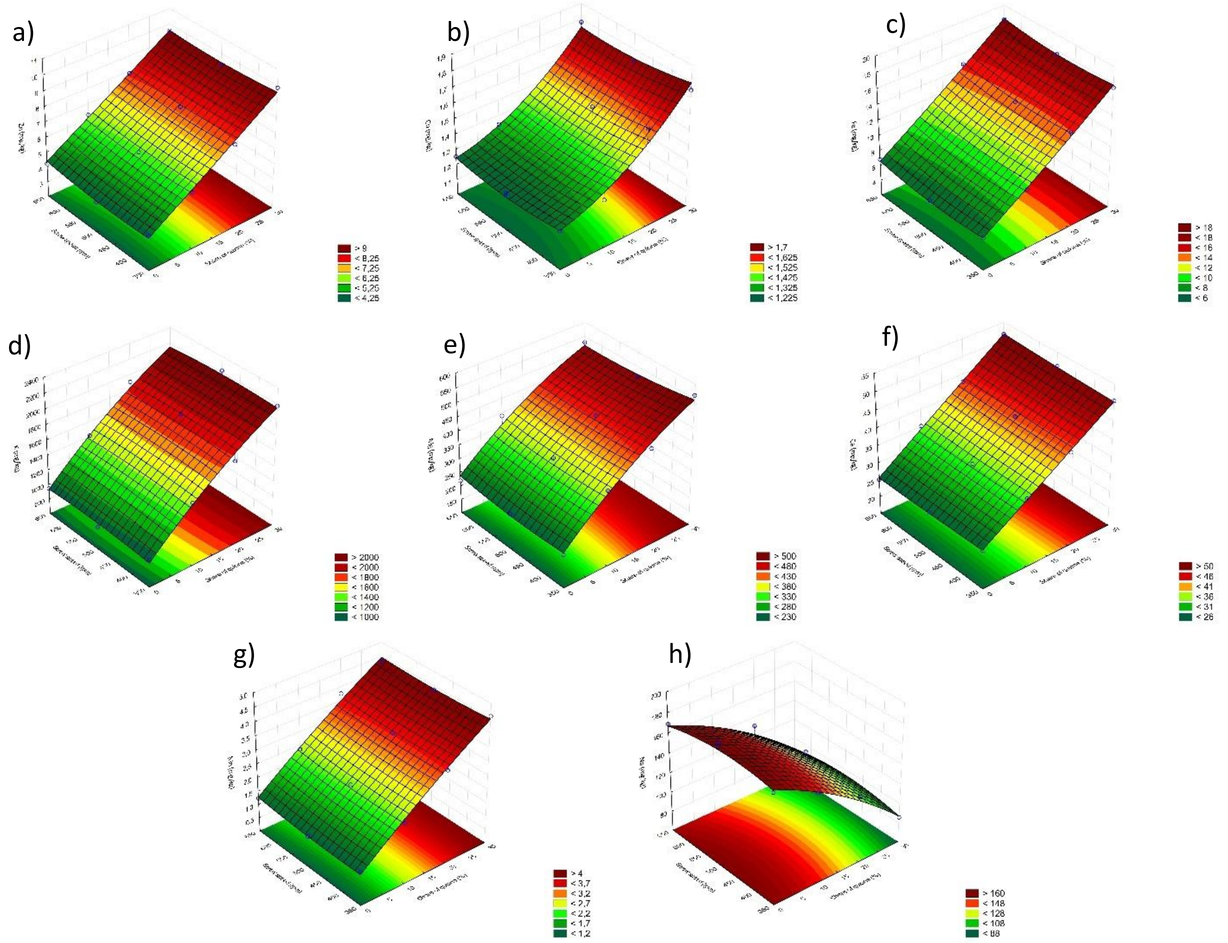

Figure S4. Graphical presentation of mathematically modeled dependance of extruded products mineral matter content from the share of quinoa and screw speed, a) Zn, b) Cu, c) Fe, d) K, e) Mg, f) Ca, g) Mg and h) Na

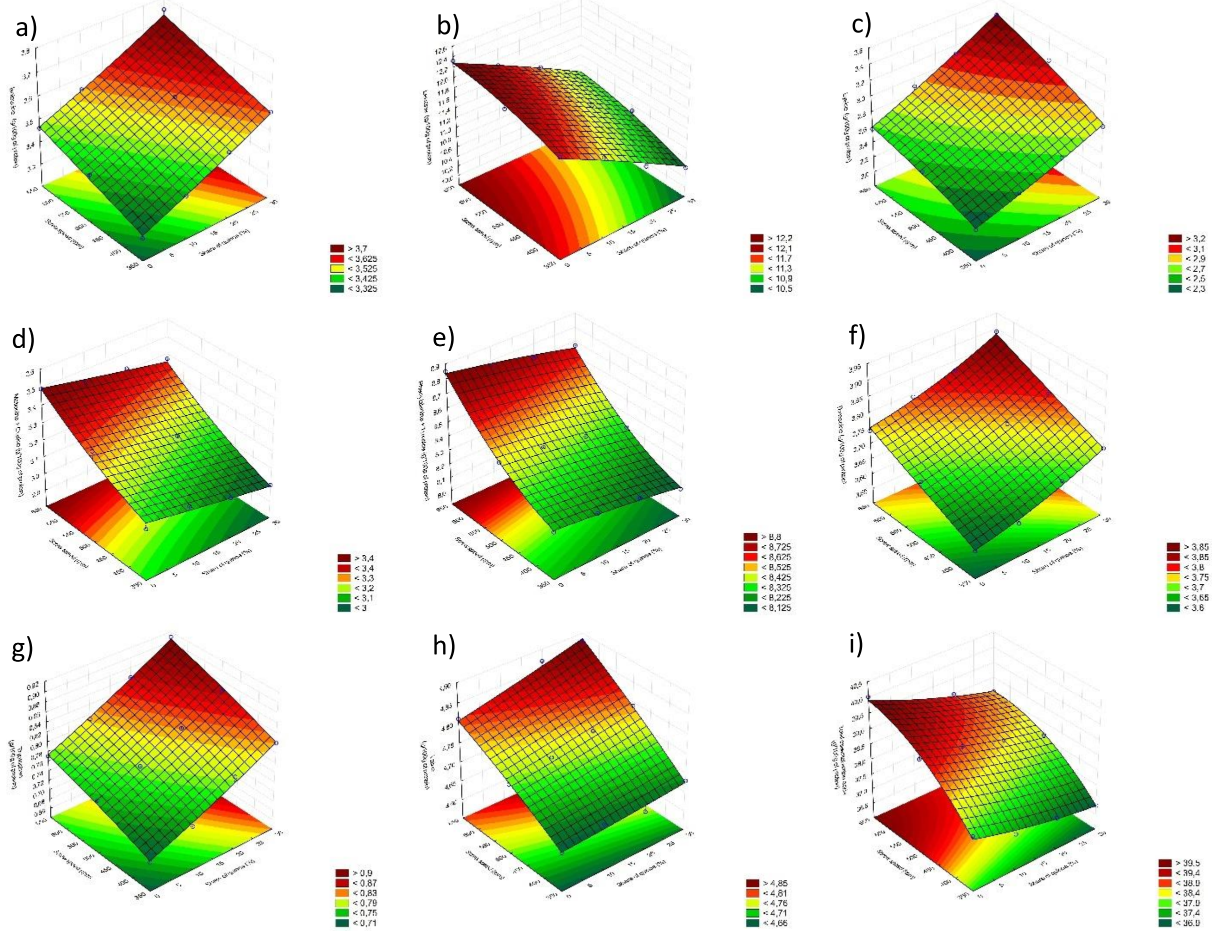

Figure S5. Graphical presentation of mathematically modeled dependance of extruded products essential aminoacids' content from the share of quinoa and screw speed, a) Isoleucine, b) Leucine, c) Lysine, d) Metionine + Cystine, e) Phenylalanine + Tyrosine, f) Threonine, g) Tryptophan, h) Valine and i) Total essental amino acids

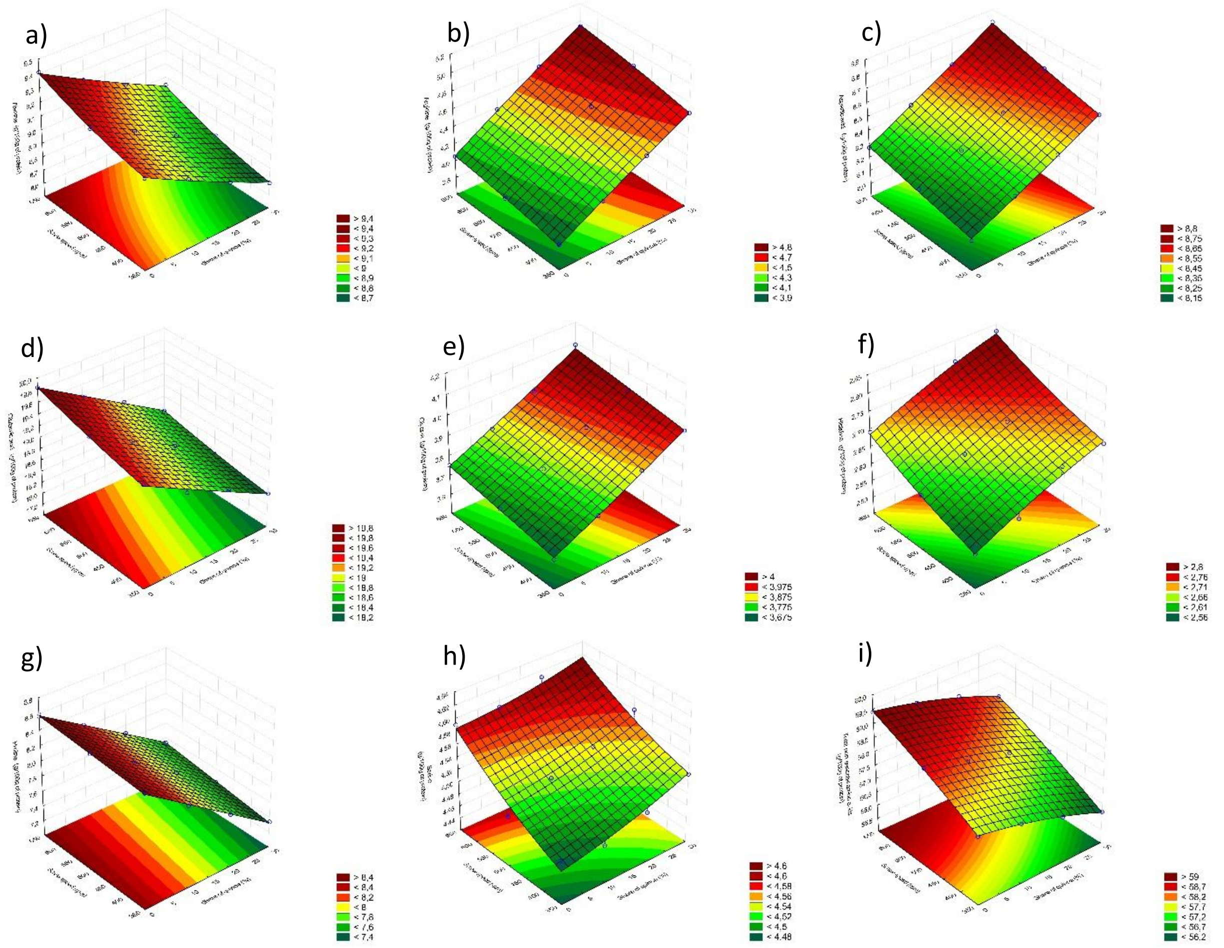

Figure S6. Graphical presentation of mathematically modeled dependance of extruded products non-essential aminoacids' content from the share of quinoa and screw speed, a) Alanine, b) Arginine, c) Aspartic acid, d) Glutamic acid, e) Glycine, f) Threonine, g) Proline, h) Serine and i) Total non-essental amino acids

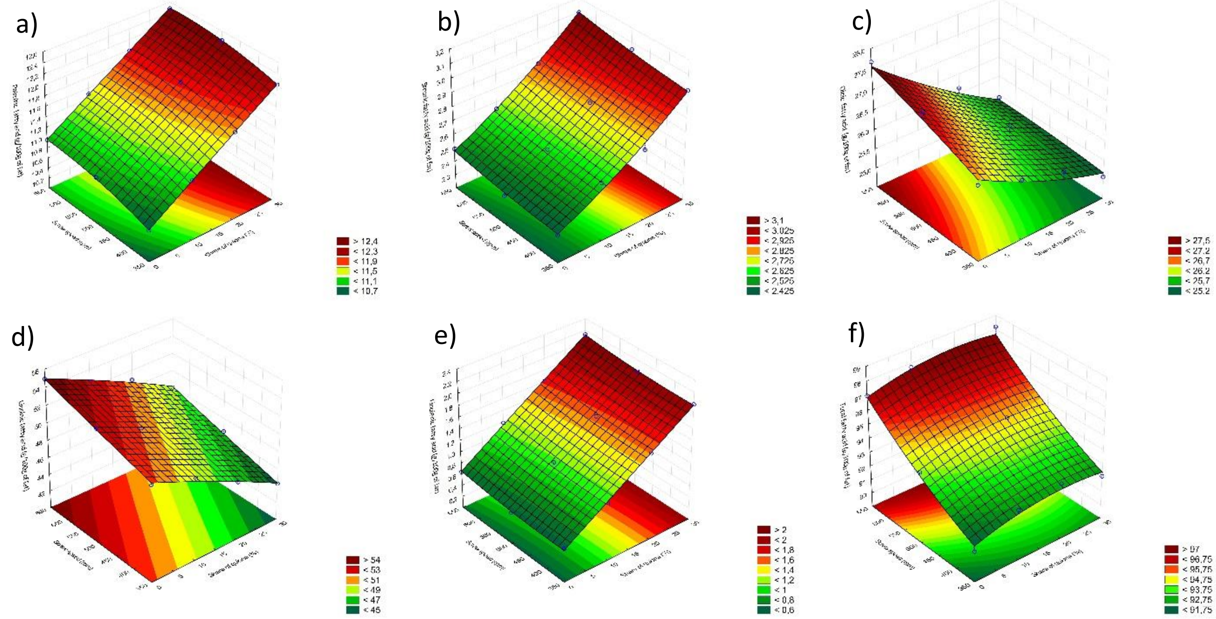

Figure S7. Graphical presentation of mathematically modeled dependance of extruded products fatty acids content from the share of quinoa and screw speed, a) Palmitic fatty acid, b) Stearic fatty acid, c) Oleic fatty acid, d) Linoleic fatty acid, e) Linolenic fatty acid and f) Total fatty acids

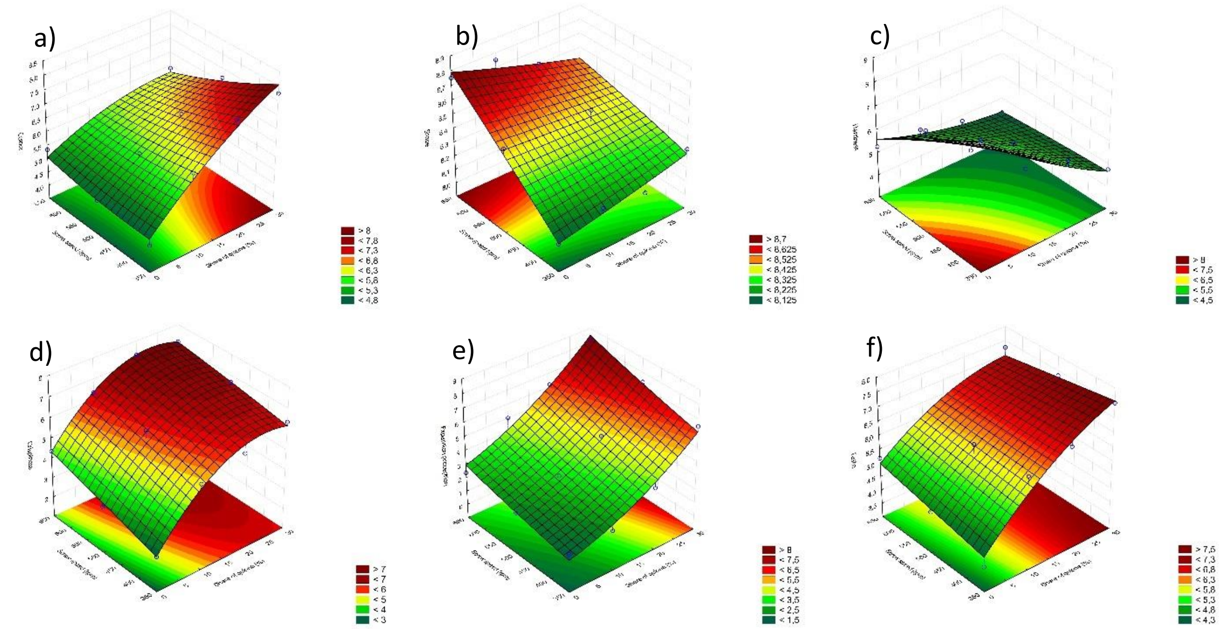

Figure S8. Graphical presentation of mathematically modeled dependance of extruded products descriptive sensory analysis from the share of quinoa and screw speed, a) Colour, b) Shape, c) Hardness, d) Crispiness, e) Expansion perception and f) Taste
